# Revealing the transcriptional heterogeneity of organ‐specific metastasis in human gastric cancer using single‐cell RNA Sequencing

**DOI:** 10.1002/ctm2.730

**Published:** 2022-02-20

**Authors:** Haiping Jiang, Dingyi Yu, Penghui Yang, Rongfang Guo, Mei Kong, Yuan Gao, Xiongfei Yu, Xiaoyan Lu, Xiaohui Fan

**Affiliations:** ^1^ Department of Medical Oncology The First Affiliated Hospital Zhejiang University School of Medicine Hangzhou China; ^2^ Pharmaceutical Informatics Institute College of Pharmaceutical Sciences Zhejiang University Hangzhou China; ^3^ Department of Pathology The First Affiliated Hospital Zhejiang University School of Medicine Hangzhou China; ^4^ Department of Gastro‐Intestinal Surgery The First Affiliated Hospital Zhejiang University School of Medicine Hangzhou China; ^5^ Department of Surgical Oncology The First Affiliated Hospital Zhejiang University School of Medicine Hangzhou China; ^6^ State Key Laboratory of Component‐Based Chinese Medicine Innovation Center in Zhejiang University Hangzhou China; ^7^ Westlake Laboratory of Life Sciences and Biomedicine Hangzhou China

**Keywords:** gastric cancer, HLA‐E‐KLRC1/KLRC2, metastasis, single‐cell RNA sequencing, tumoural heterogeneity

## Abstract

**Background:**

Deciphering intra‐ and inter‐tumoural heterogeneity is essential for understanding the biology of gastric cancer (GC) and its metastasis and identifying effective therapeutic targets. However, the characteristics of different organ‐tropism metastases of GC are largely unknown.

**Methods:**

Ten fresh human tissue samples from six patients, including primary tumour and adjacent non‐tumoural samples and six metastases from different organs or tissues (liver, peritoneum, ovary, lymph node) were evaluated using single‐cell RNA sequencing. Validation experiments were performed using histological assays and bulk transcriptomic datasets.

**Results:**

Malignant epithelial subclusters associated with invasion features, intraperitoneal metastasis propensity, epithelial–mesenchymal transition‐induced tumour stem cell phenotypes, or dormancy‐like characteristics were discovered. High expression of the first three subcluster‐associated genes displayed worse overall survival than those with low expression in a GC cohort containing 407 samples. Immune and stromal cells exhibited cellular heterogeneity and created a pro‐tumoural and immunosuppressive microenvironment. Furthermore, a 20‐gene signature of lymph node‐derived exhausted CD8^+^ T cells was acquired to forecast lymph node metastasis and validated in GC cohorts. Additionally, although anti‐NKG2A (KLRC1) antibody have not been used to treat GC patients even in clinical trials, we uncovered not only malignant tumour cells but one endothelial subcluster, mucosal‐associated invariant T cells, T cell‐like B cells, plasmacytoid dendritic cells, macrophages, monocytes, and neutrophils may contribute to HLA‐E‐KLRC1/KLRC2 interaction with cytotoxic/exhausted CD8^+^ T cells and/or natural killer (NK) cells, suggesting novel clinical therapeutic opportunities in GC. Additionally, our findings suggested that PD‐1 expression in CD8^+^ T cells might predict clinical responses to PD‐1 blockade therapy in GC.

**Conclusions:**

This study provided insights into heterogeneous microenvironment of GC primary tumours and organ‐specific metastases and provide support for precise diagnosis and treatment.

## Graphical abstract

1) Single‐cell RNA‐seq of organ‐specific metastasis (liver, peritoneum, ovary, lymph node) in GC was conducted.

2) Single‐cell analysis determined phenotypes and functions of four malignant epithelial subtypes in GC.

3) A 20‐gene signature of lymph node‐derived exhausted CD8^+^ T cells might forecast lymph node metastasis, and targeting HLA‐E‐KLRC1/KLRC2 signaling severs as a novel clinical therapeutic opportunity for GC.

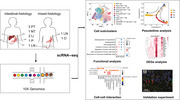

## 1 INTRODUCTION

Gastric cancer (GC) is the fourth‐most diagnosed cancer and second‐most common primary cause of cancer death worldwide, with 1.03 million new diagnoses and 720 000 deaths every year.^1^ Asia has the highest GC incidence with >70% of global cases, and China contributes most to this burden.^1^ Most patients with GC are diagnosed at advanced and metastatic stages, with diseases that are often resistant to drug therapy, and show inadequate improvement with surgical treatment, leading to low survival rates.^2^ Metastasis in different organs is the most difficult and urgent issue facing GC diagnosis and treatment. The lymph nodes, liver, peritoneum, lung, bone, and ovary are common targets, wherein tumour cells can invade distant sites via the blood, lymphatic system, and intraperitoneal spread.^3^ This distinct organ‐tropism in GC metastases is influenced by the intrinsic properties of tumour cells and the features of host regions.^4^ In metastasis process, tumour cells will evolve fitness and exhibit tropism toward to the specific organ by activation of particular genes and pathways, while the primary and host microenvironment such as the immune cells recruited by tumour also play a pivotal role in promoting metastasis progression.^4^, ^5^, ^6^ Therefore, precise mapping of the organ‐specific metastatic features is of critical importance, especially for enabling development of specific therapeutic strategies for different metastases as well as the identification of potential biomarkers for clinical diagnosis. However, the characteristics of different organ‐tropism metastases of GC are largely unknown.

Since transcriptome profiling is widely adopted to explore organ‐tropism metastasis, several groups have employed bulk RNA sequencing (RNA‐seq) to identify important genes that mediate metastasis to specific sites or whose expression in primary tumours correlates with metastatic recurrence in GC.^7^, ^8^ For instance, Zhang et al. mined the bulk RNA‐seq data from The Cancer Genome Atlas (TCGA) and identified a 28‐gene signature between lymphatic and non‐lymphatic metastases^7^; Xie et al. identified *BATF2* as a downregulated gene associated with peritoneal recurrence after curative gastrectomy in GC.^8^ Nonetheless, these bulk methods cannot detect the cell diversity and obscure the intra‐ and inter‐tumoural complexity in GC.

Recently, single‐cell RNA sequencing (scRNA‐seq) technology has enabled accurate and in‐depth studies of intra‐ and inter‐tumoural heterogeneity in various cancers.^9^ Several single‐cell signatures across inflammation, premalignant lesions and early GC have been revealed, including biomarkers of gastric early malignant cells.^9^, ^10^, ^11^ Rare tumour types, intratumour subclones and widespread reprogramming in the tumour microenvironment (TME) of primary GC were identified based on scRNA‐seq, supporting the heterogeneity of GC.^6^, ^10^ More importantly, such single‐cell resolution analysis revealed the features of GC peritoneal dissemination by investigation of peritoneal carcinomatosis.^12^, ^13^ Specifically, Yasuda et al. showed that inflammation driven senescent cancer‐associated fibroblasts enhance the peritoneal dissemination through JAK/STAT3 signalling^12^; Wang et al. demonstrated that diversity in tumour cell lineage/state compositions of GC peritoneal carcinomatosis.^13^ Collectively, scRNA‐seq is a powerful and unbiased tool for analysing heterogeneous and functional subpopulations. Herein, we investigated primary tumours and different metastases (liver, peritoneum, ovary, lymph node) of GC to examine the intra‐ and inter‐tumoural heterogeneity of carcinoma cells and TME and to better understand the different organ metastatic patterns with a special focus on lymphatic metastases using scRNA‐seq.

## 2 RESULTS

### 2.1 Single‐cell transcriptional landscape of primary and metastatic GC

**FIGURE 1:**
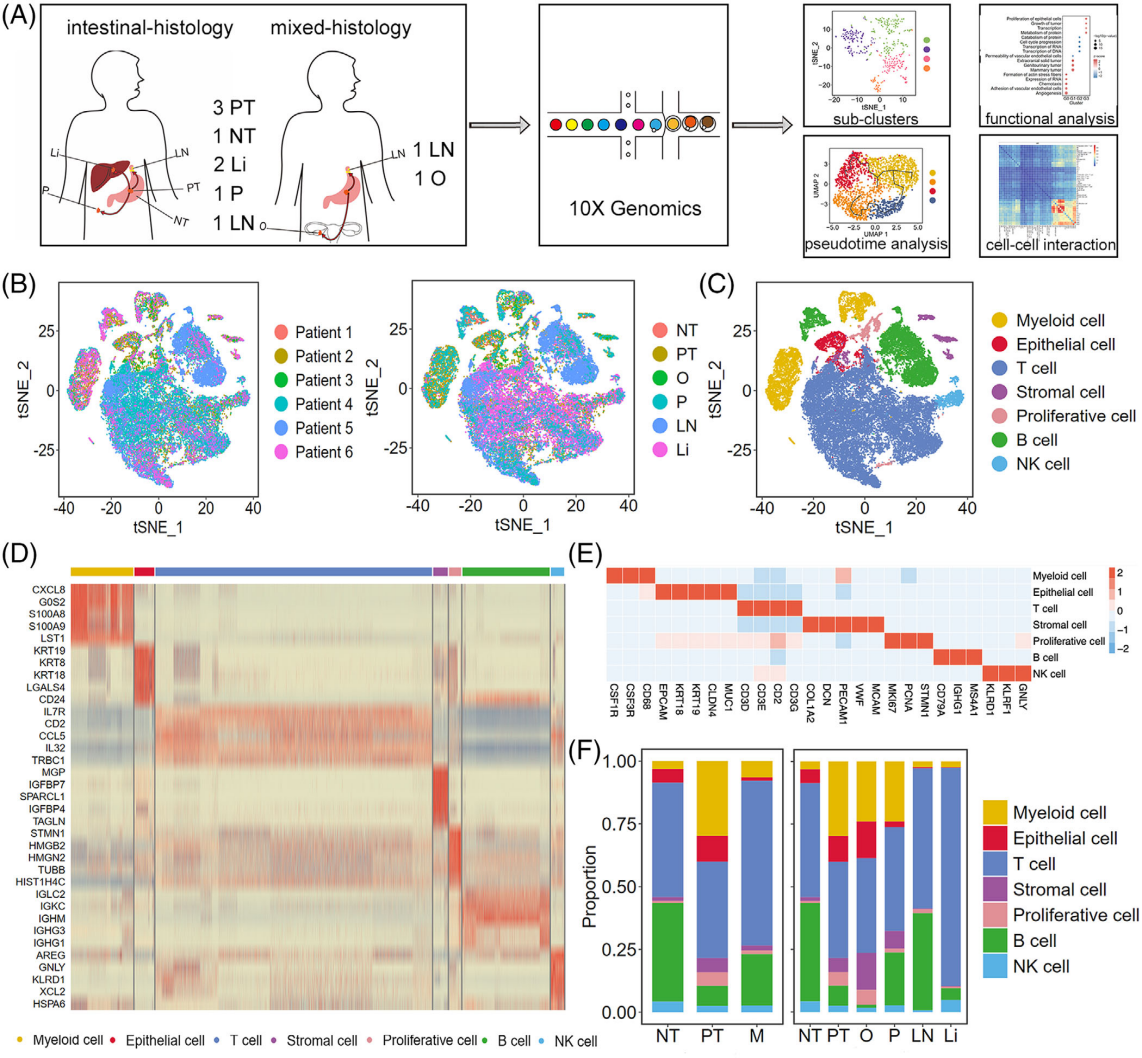
Single‐cell transcriptome profiles of GC primary tumour, metastasis and adjacent non‐tumour samples. (A) scRNA‐seq and data analyses. Ten fresh human tissue samples from six patients were collected, including three primary GC samples (PT; i.e., PT1, PT2, and PT3), one adjacent non‐tumour sample (NT; i.e., NT1) and six metastasis samples (M). For the metastases, we obtained two liver tumour samples (Li; i.e., Li1, Li2), two metastatic lymph nodes samples (LN; i.e., LN1, LN2), one peritoneal tumour sample (P; i.e., P1), and one ovary tumour sample (O; i.e., O1) during gastroscopy, biopsy, or surgical resection. (B) t stochastic neighbour embedding (tSNE) projection within each patient and sample origin. (C) tSNE showing seven cell types for the 42 968 cells. (D) Heatmap of highly variable genes for seven major lineages. (E) Heatmap of marker genes for seven major lineages. (F) Proportion of each cell type in NT, PT, M, Li, LN, P, and O

We performed scRNA‐seq analysis of 10 fresh human tissue samples from six patients, including three primary tumour samples (PT; i.e., PT1, PT2 and PT3), one adjacent non‐tumoural sample (NT; i.e., NT1), and six metastatic samples (M). Among M, we obtained two liver metastasis samples (Li; i.e., Li1 and Li2), two lymph nodes metastasis samples (LN; i.e., LN1 and LN2), one peritoneal metastasis sample (P; i.e., P1), and one ovary metastasis sample (O; i.e., O1) during gastroscopy, biopsy or surgical resection (Figure 1A and Table S1). PT1 and Li1 were from Patient 1; PT2 and NT1 were from Patient 2; O1 was from Patient 3; PT3, Li2 and LN1 were from Patient 4; LN2 was from Patient 5; and P1 was from Patient 6. PT1, PT2, PT3, Li1, Li2, LN1, and P1 were intestinal‐histology GC samples, whereas LN2 and O1 were mixed‐histology GC samples. Enrolled patients were diagnosed recently and had not been administered therapy. After quality filtering, 42 968 cells were detected, with a median of 1639 genes per cell, among which 12 014, 28 653 and 2301 cells were collected from PT, M and NT, respectively. After dimensionality reduction and unsupervised cell clustering, we identified epithelial (1743; *EPCAM*, *KRT19*, *CLDN4*), stromal (1288; *PECAM1*, *CLO1A2*, *VWF*), proliferative (1089; *MKI67*, *STMN1*, *PCNA*),^14^, ^15^, ^16^ T (24 448; *CD3D*, *CD3E*, *CD2*), B (7708; *CD79A*, *IGHG1*, *MS4A1*), natural killer (NK, 1173; *KLRD1*, *GNLY*, *KLRF1*), and myeloid (5519; *CSF1R*, *CSF3R*, *CD68*) cells as seven distinct lineages based on marker gene expression (Figures 1B–E and S1A). Myeloid cells included two distinct clusters, namely neutrophils and mononuclear phagocyte system cells (Figure S1B), whereas the two distinct B‐cell clusters were plasma and other B cells (Figure S1C). Owing to the different dissociation efficiency of different cell types,^17^, ^18^ immune cells accounted for the largest proportion (90.4%) among all samples, particularly in the lymph node and liver (Figure S1A), which was also observed in other scRNA‐seq data of cancer.^6^, ^9^, ^10^, ^17^, ^19^ The proportion of each cell lineage varied highly among different primary tumours and metastases (Figures 1F and S1A), revealing a heterogeneous cellular status. In addition, we compared our adjacent non‐tumoural sample to another three paired GC normal tissues^6^ and showed that NT1 was highly correlated with the three GC normal tissues (*R* = .93), indicating that our adjacent non‐tumoural sample could represent GC health cells (Figure S2A and B).

### 2.2 Four subclusters of malignant epithelial cells and relationship between their characteristics and metastasis and survival

**FIGURE 2:**
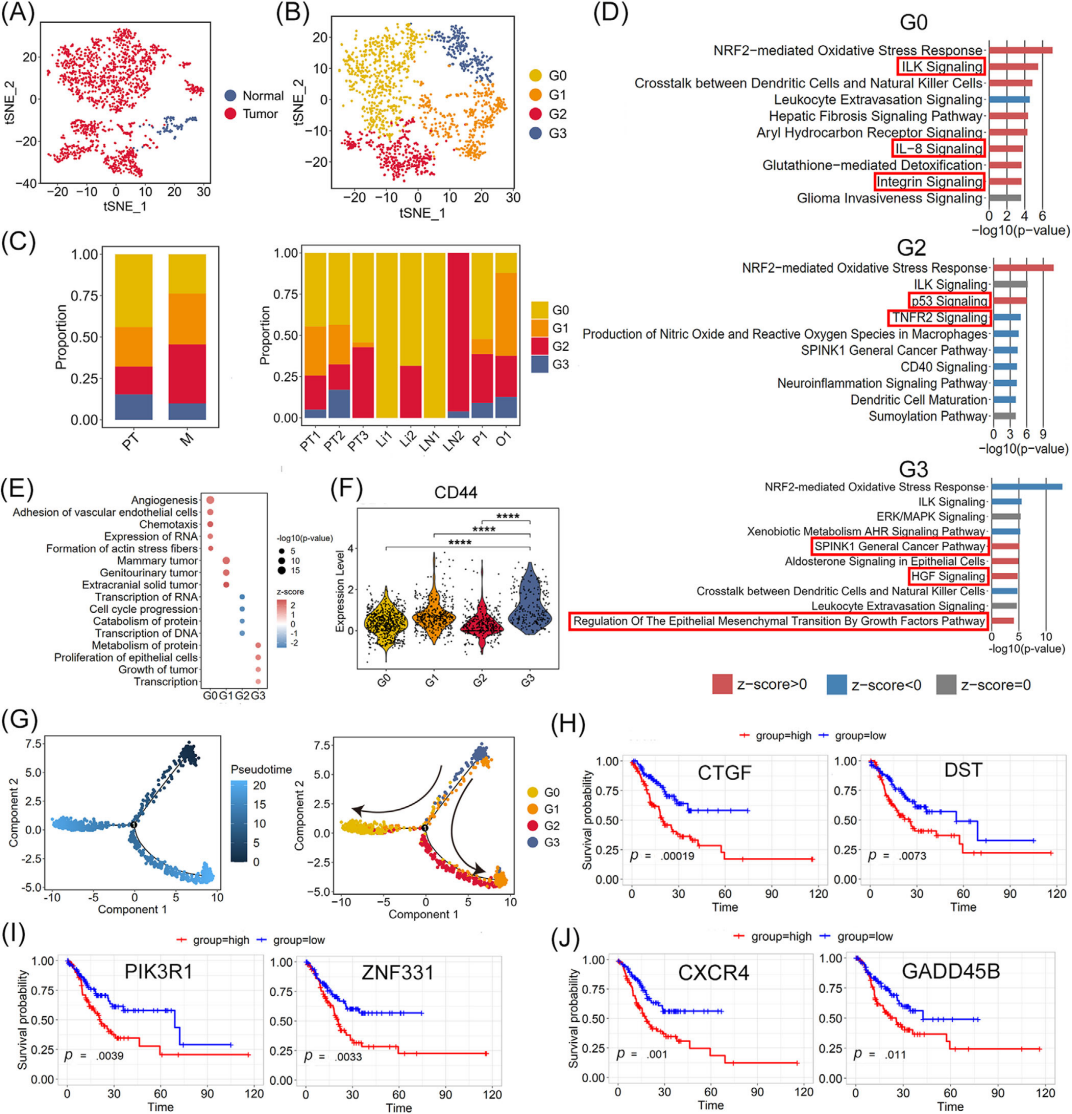
Four subclusters of malignant epithelial cells in GC and their characterisation associated with metastasis. (A) tSNE of 1615 malignant and 128 non‐malignant epithelial cells. (B) tSNE of the four malignant epithelial clusters G0–G3. (C) Relative proportion of G0–G3 cells in PT (PT1, PT2, PT3), M, Li (Li1, Li2), LN (LN1, LN2), P (P1) and O (O1). (D) IPA results showing the top enriched canonical pathways in G0, G2 or G3 cells based on the DEGs. z‐score > 0 indicates that the pathway was activated; z‐score < 0 indicates that the pathway was inhibited; z‐score = 0 indicates that the pathway was neither activated nor inhibited. (E) Dot plot showing the diseases and bio functions of G0–G3 cells based on DEGs using IPA analysis. (F) Violin plot showing the expression of *CD44* in G0–G3 cells. ^****^ *p* < .0001. (G) Unsupervised transcriptional trajectory of G0–G3 subsets predicted by Monocle 2. (H–J) High level of G0‐, G1‐ and G3‐associated genes predicted poor prognosis in the TCGA‐STAD.htseq_counts.tsv dataset (*n* = 407 patients). Log‐rank *p* < .05 was considered statistically significant

Malignant (1615 cells) and non‐malignant (128 cells) epithelial cells were defined using the malignant and non‐malignant scores^10^; 83% of non‐malignant epithelial cells were derived from NT (Figures 2A and S3A). Moreover, in malignant epithelial cells, the expression of several tumour‐specific genes (*CLDN4*, *CLDN7*, *TFF3*; *p *< 2.22 × 10^–16^) was significantly upregulated compared with that in the non‐malignant epithelium, whereas non‐malignant epithelial cells strongly expressed several genes associated with gastric mucus and digestive enzyme secretion (*MUC5AC*, *GKN1*, *PGC*, *LIPF*; *p* < 7.4 × 10^–9^, Figure S3B).

Clustering analysis of malignant cells in PT and M revealed four subclusters, G0–G3 (Figure 2B). Transcriptional heterogeneity in malignant cells was detected in the different samples as well as the different metastases (Figures 2C and S3C). G0 composed the main type of PT and most M (Figures 2C and S3C). Upon Ingenuity pathway analysis (IPA), G0 cells showed characteristics of tumour vascularisation and invasion, based on the differentially expressed genes (DEGs) between G0 and the other malignant epithelia. Specifically, several pathways, including angiogenesis and adhesion of vascular endothelial cells, and formation of actin stress fibres, were significantly enriched, based on diseases and bio functions analysis in IPA (Figure 2E). Integrin signalling, which might mediate cell–matrix contact and govern tumour invasive and metastatic potential,^20^ was positively regulated in G0 cells (Figure 2D). Vascular endothelial growth factor A (*VEGFA*), the key factor in angiogenesis, was markedly upregulated in G0 cells and activated in the ILK pathway and IL‐8 signalling (Figure 2D and Table S2). Other genes related to cell invasion and angiogenesis, such as *ICAM‐1*, *FOS*, *ITGB1*, and *ITGAV*, were also upregulated in the aforementioned pathways (Table S2). Therefore, G0 cells may have angiogenic and invasive properties. G1 cells specifically expressed the epithelial–mesenchymal transition (EMT)‐related genes *SRGN*, *VIM*, and *LAPTM5A* (Figure S3D) and were found mainly in O and P (Figure 2C). IPA showed that the pathways of mammary tumours, genitourinary tumours and extracranial solid tumours were strongly activated in G1 cells, in which *AREG*, *ARL4C*, and *ARID5B* showed the greatest contribution to activation (Figure 2E and Table S3). *AREG* and *ARID5B* can promote cell proliferation and migration in GC,^21^, ^22^ and *ARL4C* is closely associated with peritoneal dissemination.^23^ Thus, migration and EMT are the main characteristics of G1 cells, and these cells are more likely to migrate via intraperitoneal spread. G2 cells were predominantly found in one LN (LN2) and PT3 (Figure 2C). p53 signalling was activated in G2 cells (Figure 2D), which would induce apoptosis and suppress tumour growth. Marked downregulation of *JUN*, *PIK3R1*, and *MDM2* and upregulation of *CDKN2A* (*P19ARF*) were observed in this pathway (Figure 2D and Table S2), which might have decreased proliferation and invasion in GC.^24^, ^25^ Thus, G2 cells might be dormancy‐like tumour cells in GC. G3 cells were mainly found in the PT (PT1, PT2) and M (O1, P1, LN2), except in the Li (Li1, Li2). The SPINK1 general cancer pathway and HGF signalling were activated in G3 cells in which the tumour malignancy‐related genes *RASD1*, *PIK3R1*, *JUN*, and *FOS* were significantly upregulated, indicating that G3 promotes malignant progression in GC (Figure 2D and Table S2). Regulation of EMT by the growth factor pathway was also significantly enriched (Figure 2D) and EMT‐related genes (*ZEB2*, *VIM* and *ID2*) were markedly upregulated in these pathways, indicating that EMT progression is induced in G3 cells (Table S2). The expression of *CD44*, *PROM1* (*CD133*), *LGR5*, *SOX2*, *TFRC* (*CD71*), *CXCR4*, and *JAG1*, markers of cancer stem cells (CSCs),^26^, ^27^, ^28^, ^29^ was also significantly upregulated in G3 compared with that in the other subclusters of malignant epithelial cells (Figures 2F and S4A). CSCs play an important role in cancer cell proliferation, migration and metastasis.^30^ Thus, we calculated the proliferation and migration scores for subclusters G0–G3 based on the expression of genes related to tumour cell proliferation (*MKI67*, *IGF1*, *ITGB2*, *PDGFC*, *JAG1*, and *PHGDH*)^31^, ^32^ or migration (*VIM*, *SNAI1*, *MMP9*, *AREG*, *ARID5B*, and *FAT1*).^21^, ^22^, ^33^, ^34^ As shown in Figure S4B and C, G3 cells showed the highest proliferation and migration scores compared with the other subclusters, suggesting that G3 cells have an EMT‐induced CSC phenotype. Consistently, the pseudotime trajectory axis derived from Monocle 2 showed the dynamic characteristics and heterogeneity of malignant epithelial cells (Figures 2G and S3E). Specifically, G3 cells with the EMT‐induced CSC phenotype were observed in the initial part followed by G1 cells, which exhibited characteristics of EMT and migration. Dormancy‐like tumour G2 and proangiogenic and invasive G0 cells were located in separate trajectory branches, suggesting distinct differentiation states.

Survival analysis of cancer cell‐associated genes was performed using a stomach adenocarcinoma cohort from TCGA dataset (TCGA‐STAD.htseq_counts.tsv dataset, *n* = 407). Patients with GC showing high expression of G0, G1 and G3‐associated genes (*DST*, *CTGF*; *PIK3R1*, *ZNF331*; *CXCR4*, *GADD45B*) exhibited worse overall survival (*p* < .05) than those with low expression (Figures 2H–J and S3F–H). Furthermore, the clinical data verified that patients with ovarian metastasis (Patient 3) with a higher proportion of G1 and G3 cells had poor clinical prognosis (Table S4).

To further delineate tumour phenotype and clonal substructure, CopyKAT was applied to determine the copy number variants (CNVs) in GC primary tumour and metastases based on a previously published paper.^35^ CopyKAT can be used to delineate clonal substructure based on the genomic copy number profiles.^35^ Since GC is a typical epithelium‐originated malignant tumour, CNVs in malignant epithelial cells were investigated. Based on the differences in CNVs, four subclones (0‐3) were identified in the malignant epithelial cells (Figure S5A and B). Subclones 0 and 2 were mainly found in PT2 obtained from Patient 2 without metastases, whereas subclone 3 was found in PT1 and Li1, both of which were obtained from Patient 1 with liver metastasis, and subclone 1 was mostly derived from the remaining metastasis samples (Figure S5C). Gene Ontology (GO) analysis was performed based on the DEGs to identify phenotypic differences among the subclones. Multiple cancer hallmark pathways such as GC network, transcriptional regulation by TP53, and cell population proliferation were enriched in subclone 0 (Figure S5D), whereas antigen processing and presentation, cellular response to topologically incorrect protein, and hormone‐mediated signalling pathway were activated in subclone 2 (Figure S5E), showing the intra‐tumoural heterogeneity in the primary tumour PT2. Subclone 3 was enriched for multiple protein‐related processes, including negative regulation of protein modification process, positive regulation of protein localisation, and activation of protein kinase B activity (Figure S5F), showing unique CNVs in Patient 1 with distal gastric adenocarcinoma and liver metastasis. Subclone 1 was enriched for DNA damage pathways such as DNA damage recognition in GG‐NER and cellular response to DNA damage stimulus (Figure S5G), suggesting DNA abnormality in GC metastasis.

Furthermore, we investigated the malignant score performance for non‐epithelial cells by calculating the malignant and non‐malignant scores.^10^ Figure S6A shows that 97% of non‐epithelial cells were non‐malignant, whereas 3% of non‐epithelial cells were malignant. Most of these malignant non‐epithelial cells were found in myeloid cells, stromal cells and proliferative cells (Figure S6B and C). In line with these findings, several tumour‐related genes, such as *CCL7* and *CSF2*, used to calculate the malignancy score (Table S5 lists the genes used for calculating the malignancy score) were found to be expressed in the non‐epithelial cells in GC^36^, ^37^; this may be the reason 3% of non‐epithelial cells were malignant. Moreover, since GC is a typical epithelium‐originated malignant tumour,^38^ we detected the expression of epithelial markers on these malignant non‐epithelial cells. Compared with malignant epithelial cells, malignant non‐epithelial cells rarely expressed the epithelial marker genes *EPCAM*, *KRT18*, or *KRT19* (Figure S6D). In contrast, these malignant non‐epithelial cells expressed their own canonical marker genes, illustrating that these cells were not GC tumour cells.

### 2.3 T and B cells mediate various immune responses during GC progression

**FIGURE 3:**
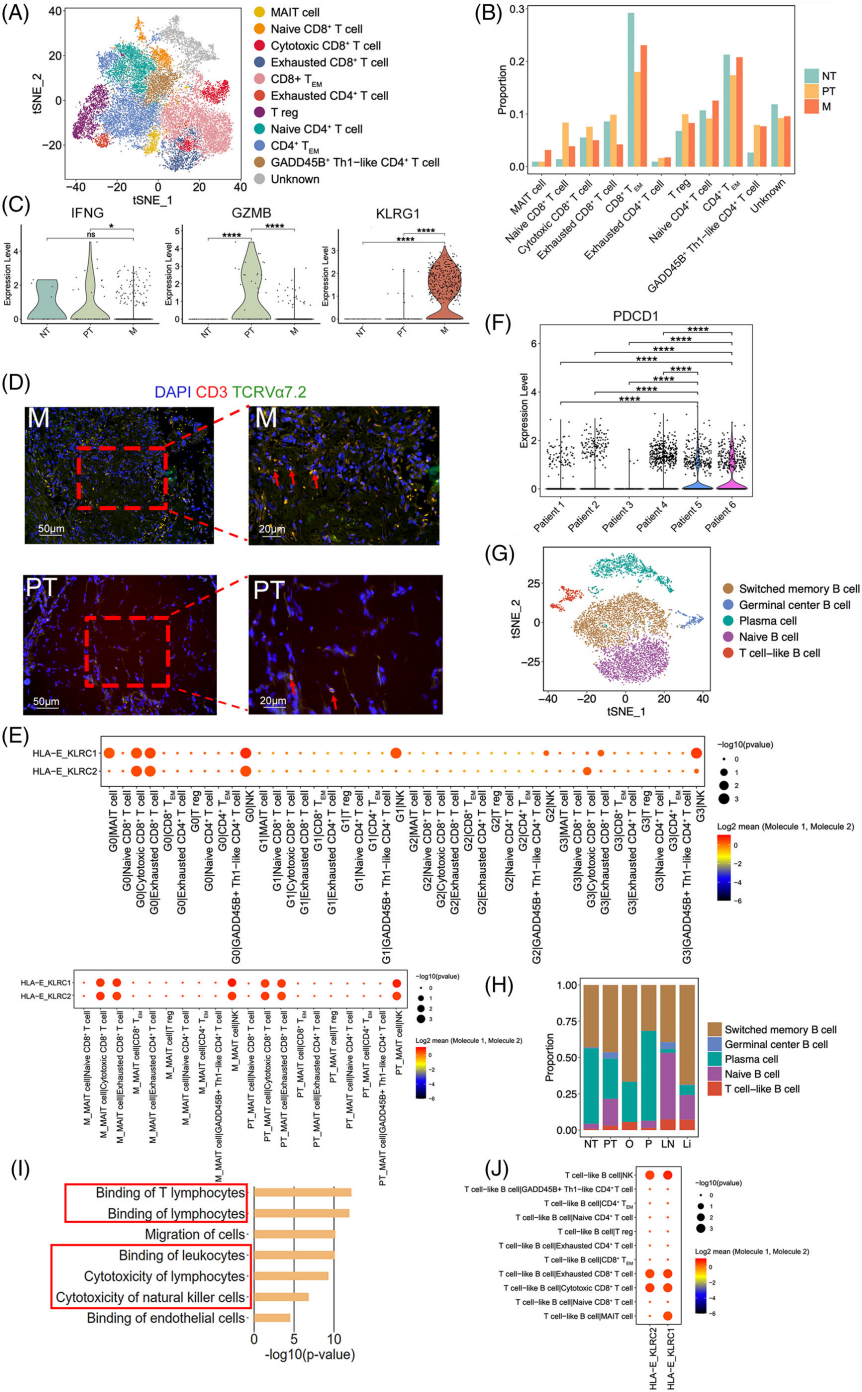
Various immune responses are mediated by T and B cells during GC progression. (A) tSNE of T cells. Tregs: regulatory T cells; CD4^+^ T_EM_: effector memory CD4^+^ T cells; GADD45B^+^ Th1‐like CD4^+^ T cells: GADD45B^+^ T helper type 1‐like CD4^+^ T cells; CD8^+^ T_EM_: effector memory CD8^+^ T cells; MAIT: mucosal‐associated invariant T. (B) Relative proportion of each T cluster in NT, PT and M. NT: NT1; PT: PT1, PT2, PT3; M: Li1, Li2, LN1, LN2, O1, P1. (C) Violin plot showing expression of *IFNG*, *GZMB*, and *KLRG1* in MAIT cells in different samples. ^*^ *p* < .05; ^***^ *p* < .001; ^****^ *p* < .0001; ns means *p* > .05. (D) Immunofluorescence staining indicates the co‐expression of CD3, TCR Vα7.2, and DAPI (nuclei) on MAIT cells in M and PT. (E) Bubble plots exhibiting significant interactions between cancer cells and T/NK cells as well as MAIT cells in M/PT and T/NK cells by the ligand–receptor pair HLE‐A‐KLRC1/KLRC2. (F) Violin plot showing the expression of *PDCD1* in CD8^+^ T cells across different patients. ^****^ *p* < .0001. (G) tSNE of B cells. (H) Relative proportion of each B cluster in NT, PT, O, P, LN, and Li. (I) IPA results showing the diseases and bio functions in T cell‐like B cells based on the DEGs. (J) Bubble plots exhibiting significant interactions between T cell‐like B cells and other cell groups by the ligand–receptor pairs HLA‐E‐KLRC1/KLRC2

T and B cells made up a large proportion of cells in all samples. T lymphocytes are involved in many different types of immune responses and targeted by many immune‐checkpoint inhibitors. In total, 24 448 T cells were subjected to unsupervised clustering to reveal subtypes. Eleven subclusters were identified with unique signature genes, including five CD8^+^ subclusters, five CD4^+^ subclusters and one unknown subcluster (Figures 3A, 3B and S7A–C). Five CD4^+^ subclusters were composed of naïve CD4^+^ T cells (*CCR7*, *LEF1*), regulatory T cells (Tregs; *CTLA4*, *FOXP3*), exhausted CD4 T cells (*CXCL13*, *TIGIT*), effector memory CD4^+^ T cells (CD4^+^ T_EM_; *CCL5*, *ANXA1*, *GZMA*) and GADD45B^+^ T helper type 1 (Th1)‐like CD4^+^ T cells (*GADD45B, TNF*).^39^, ^40^, ^41^, ^42^, ^43^, ^44^ For CD8^+^ subclusters, naïve CD8^+^ T cells (*LEF1*, *SELL*), cytotoxic CD8^+^ T cells (*GNLY*, *GZMB*), exhausted CD8^+^ T cells (*CTLA4*, *LAG3*, *TIGIT*), effector memory CD8^+^ T cells (CD8^+^ T_EM_; *GZMK*, *CXCR4, EOMES*) and mucosal‐associated invariant T (MAIT) cells (*SLC4A10*, *KLRB1*) were the main components (Figure S7D, Table S6).^39^, ^40^, ^45^ As shown in Figure 3B, tumour samples (PT and M) were enriched in exhausted CD4^+^ T cells and Tregs, whereas the proportion of CD4^+^ T_EM_, CD8^+^ T_EM_ and cytotoxic CD8^+^ T cells was slightly reduced in M compared with the level in NT as expected,^46^ indicating a suppressive immune microenvironment created during tumour progression and metastasis. Moreover, compared with that in NT1, the proportion of cytotoxic CD8^+^ T cells was higher in PT1 and PT2 and lower in PT3 (Figure S7C), thereby illustrating the inter‐tumoural heterogeneity. Similar phenomena were observed in the individual patients with other cancers.^47^, ^48^ In addition, more MAIT cells were found to appear in M than in PT and NT. Normal MAIT cells exhibit an effector memory T‐cell phenotype and induce cytotoxic responses^49^; however, only MAIT cells in NT and PT expressed the Th1 cytokine *IFNG*, with almost no expression detected in the MAIT cells of M (Figure 3C). Additionally, only the MAIT cells in PT highly expressed *GZMB*, which encodes a protein that can kill infected cells,^49^ with MAIT cells in M showing no expression of *GZMB* (Figure 3C). Similar results were found in the patients with colorectal and hepatocellular carcinomas, as the cytotoxic effector gene *GZMB* was highly expressed in the MAIT cells isolated from primary tumours compared with that in tumour‐adjacent normal tissues, which shows a protective immune response against tumours.^50^, ^51^ In contrast, MAIT cells in M highly expressed *KLRG1* (Figure 3C), which is associated with T‐cell dysfunction.^52^ Immunofluorescence staining showed that MAIT cells appeared both in PT and M, with a higher number in M (Figure 3D).

To verify the characteristics of T‐cell subclusters and decipher the molecular relationship between them and other cell subclusters, a crosstalk network was constructed using potential receptor–ligand pair interactions. A strong cell–cell interaction (CCI) between cancer subclusters (G0, G1 and G3) and cytotoxic/exhausted CD8^+^ T cells and/or NK cells was predicted based on the HLA‐E‐KLRC1/KLRC2 pair (Figure 3E). HLA‐E‐KLRC1 serves as a novel checkpoint and functions as an acquired resistance mechanism in the TME.^53^ The expression level of *KLRC1*/*KLRC2* was detected in cytotoxic/exhausted CD8^+^ T and NK cells. NK cells showed the highest expression of *KLRC1*, whereas cytotoxic CD8^+^ T cells exhibited the highest expression of *KLRC2*, and exhausted CD8^+^ T cells displayed moderate expression of these two genes (Figure S7E). These observations are in line with previous findings.^54^, ^55^, ^56^, ^57^, ^58^ NKG2A (*KLRC1*), an immune inhibitor receptor, had been observed on NK and T cells.^54^ In TME, more than half the NK cells have been shown to express *KLRC1*,^54^, ^55^ with a substantial fraction of intratumoural CD8^+^ T cells showing upregulated *KLRC1*, especially those harboring tissue‐resident signature or the exhausted CD8^+^ T cells.^55^, ^56^ NKG2C (*KLRC2*) has also been investigated on NK cells, natural killer T (NKT) cells, γδ T cells, and CD8^+^ αβ T cells.^57^, ^58^ Although data on NKG2C in tumour are limited, *KLRC2* was identified as one of the protective genes in lower‐grade gliomas,^58^ which was in line with its high expression in the cytotoxic CD8^+^ T cells in this study. CCI analysis also predicted that the ligand and receptor pair HLA‐E‐KLRC1 is involved in the interaction between MAIT cells in M/PT and cytotoxic/exhausted CD8^+^ T cells as well as that between MAIT cells in M/PT and NK cells (Figure 3E).

The expression of multiple inhibitory receptors on T cells before treatment might help to predict the outcome of checkpoint blockade therapy in patients with cancer. Thus, we further correlated the expression of *PDCD1* in CD8^+^ T cells with the response and clinical outcome. Patients 6 (P1) and 5 (LN2) exhibited high expression of *PDCD1*, whereas others expressed low *PDCD1* expression in CD8^+^ T cells (Figure 3F). Upon tracking the case (Table S4), Patient 3 (O1), who exhibited low levels of *PDCD1* in CD8^+^ T cells, showed no improvement after using a PD‐1 inhibitor, camrelizumab, combined with paclitaxel‐albumin and tegafur for two periods, although the patient had a programmed cell death ligand 1 (*PD‐L1*) combined positive score (CPS, ratio of the number of all *PD‐L1*‐expressing cells to the number of all tumour cells) ≥ 1. Further, Patient 6, who expressed high levels of *PDCD1* in CD8^+^ T cells, showed a better response after PD‐1 inhibitor (camrelizumab) and paclitaxel‐albumin combined therapy. Immune‐checkpoint molecules PD‐1, LAG3, CTLA4, TIGIT, and HAVCR2 (TIM‐3) have been identified and studied in various cancers including liver hepatocellular carcinoma, lung adenocarcinoma, stomach adenocarcinoma, ovarian cancer, and peritoneal carcinoma.^59^, ^60^ These immune‐checkpoint molecules have been reported in the different metastatic organs of GC.^61^, ^62^ The expression of these immune‐checkpoint molecules in CD8^+^ T cells in the samples from the same patients was similar (Figure S8 and Table S7). PT1 and Li1 were from Patient 1, and both samples showed *LAG3* and *TIGIT* expression in CD8^+^ T cells, with PT1 also showing *HAVCR2* expression in CD8^+^ T cells. PT2 and NT1 were from Patient 2, and both expressed *LAG3* and *TIGIT* in CD8^+^ T cells, whereas PT2 also showed *CTLA4* expression in CD8^+^ T cells. PT3, Li2, and LN1 were from Patient 4 where only Li2 showed expression of *TIGIT* in CD8^+^ T cells, whereas PT3, Li2, and LN1 did not show expression of other immune‐checkpoint molecules in CD8^+^ T cells. Patients 3, 5 and 6 only had one sample; nonetheless, Patient 6 showed expression of all five immune‐checkpoint molecules in CD8^+^ T cells, Patient 5 showed expression of *PDCD1*, *LAG3*, and *TIGIT* in CD8^+^ T cells, whereas Patient 3 did not expression of these immune‐checkpoint molecules in CD8^+^ T cells. These data indicated that Patients 1, 2, 5, and 6 might benefit from combinational blockade of specific co‐inhibitory receptors, such as TIGIT and LAG3.

Next, we clustered 7708 B cells into five cell lineages annotated using marker gene expression (Figures 3G and S7F). The proportion of each B‐cell type varied greatly between different samples (Figures 3H and S7G), indicating the heterogeneity of humoral immunity in GC primary tumours and different metastases. A special group of B cells, expressing both the typical surface markers *CD79A*, *MS4A1* (*CD20*), *CD19*, *CD40* of B lymphocytes and the marker *CD3D* of T lymphocytes,^63^, ^64^ were named the T cell‐like B cells and detected among most samples (Figures 3H, S7G and S7H). The proportion of these cells was the highest in M and lowest in NT (Figures 3H and S7G). The T cell‐like B cells in GC was further confirmed in the metastatic lymph node LN2 and two new GC metastases we obtained (LN3 from the patient who had GC with lymph node metastasis; O2 from the patient who had GC with ovary metastasis) with the marker genes *CD19* and *CD3D* (Figure S9). Pathway analysis of T cell‐like B cell‐associated DEGs revealed that binding of leukocytes/lymphocytes, and cytotoxicity of NK cells/lymphocytes were significantly enriched, indicating the interaction of T cell‐like B cells and these clusters (Figure 3I). CCI analysis showed that the ligand and receptor pair HLA‐E‐KLRC1/KLRC2 was predicted to be involved in the interaction between T cell‐like B cells and NK cells and between T cell‐like B cells and cytotoxic/exhausted CD8^+^ T cells (Figure 3J).

### 2.4 Endothelial cells promote angiogenesis and create immune resistance

**FIGURE 4:**
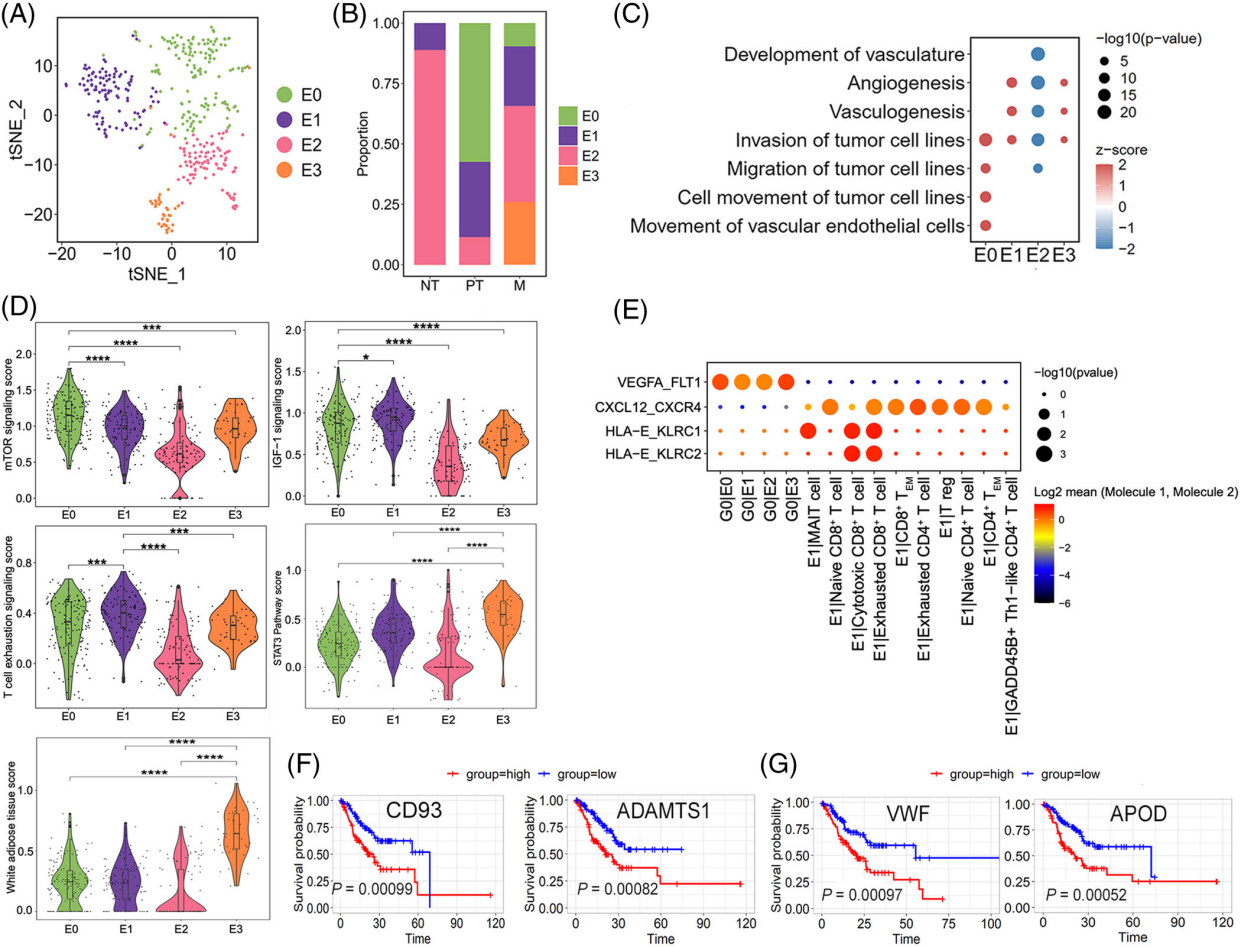
Endothelial cells promote angiogenesis and create immune resistance in GC. (A) tSNE of the four endothelial cell clusters E0–E3. (B) Relative proportion of E0–E3 from NT, PT, and M. (C) Dot plot showing the diseases and bio functions of E0–E3 based on DEGs by IPA. (D) Violin plots showing the pathway scores of top enriched canonical pathways (IPA) in E0–E3 based on the DEGs. ^*^ *p* < .05; ^***^ *p* < .001; ^****^ *p* < .0001. (E) Bubble plots exhibiting significant interactions between endothelial cells and other cell groups by ligand–receptor pairs. (F–G) High levels of E0‐ and E3‐associated genes predicted poor prognosis in the TCGA‐STAD.htseq_counts.tsv dataset (*n* = 407 patients). Log‐rank *p* < .05 was considered statistically significant

In this study, 399 cells were identified as endothelial cells with concordant expression of canonical marker genes (Figure S10A). Subclustering of endothelial cells revealed four clusters, E0–E3 (Figures 4A and S10B). E0 cells specifically expressed *IGFBP5*, *STC1*, and *IGFBP3* were found in PT and M but not in NT (Figures 4B, S10B and C). *IGFBP3* and *STC1* influence angiogenic sprouting; proangiogenic *VEGFA* and *TGF‐β* are upstream regulatory factors of *IGFBP5*. According to IPA, both mTOR and IGF‐1 signalling, which are tightly related to tumour invasion in GC,^65^ were positively regulated in E0 cells, and diseases and biofunctions analysis showed that E0 cells may increase the invasion and migration of tumour cells (Figure 4C and D), indicating that E0 cells promote GC invasion. E1 cells were most abundant in PT and M (Figure S10C). IPA also showed that E1 cells were closely associated with the regulation of the T‐cell exhaustion signalling pathway (Figure 4D). Notably, the gene expression network of the T‐cell exhaustion signalling pathway showed that *FOXO1*, *FOXP1*, and *JUN* were activated (Table S2), and these genes were related to the inhibition of CD8^+^ T‐cell effector and memory functions and induction of T‐cell unresponsiveness in cancer,^66^ supporting the notion that E1 cells suppress the immune response. Most endothelial cells in NT were E2 cells (88%), which displayed lower activity than other three subclusters, suggesting that they were normal endothelial cells (Figure 4C and D). E3 cells were found only in LN and O (Figures 4B and S10C). The white adipose tissue browning pathway was activated in E3 cells, in which the expression of the *VEGF* receptor‐encoding gene *NRP1* and fibroblast growth factor receptor‐encoding gene *FGFR1* were significantly upregulated (Figure 4D and Table S2). In addition, the STAT3 pathway was markedly activated in E3 cells (Figure 4D), which promoted invasion and lymphangiogenesis in GC cells.^67^ Functional analysis via IPA showed that E3 cells increased angiogenesis and vasculogenesis (Figure 4C). Thus, E3 cells might be associated with angiogenesis, lymphangiogenesis, and tumour cell invasion. Figure 4E shows a dominant crosstalk between endothelial cells and G0 cells through angiogenesis signalling, particularly for E0 and E3, which receive potential angiogenic stimulatory signals from angiogenic G0 cells through VEGFA and its receptor FLT1 (also known as *VEGFR1*), key promoters of angiogenesis in cancer, including GC. The ligand–receptor pair HLA‐E‐KLRC1/KLRC2 was involved in interactions between E1 and cytotoxic/exhausted CD8^+^ T cells. A strong interaction between E1 and T cells (exhausted CD4^+^ and CD8^+^ T cells) was also predicted via the CXCL12‐CXCR4 pair, which is the mechanism of immune resistance in GC^68^ and in line with the immunosuppressive feature of E1. Furthermore, we assessed the clinical impact of the characteristic signature of E0 and E3 using a stomach adenocarcinoma cohort (TCGA‐STAD.htseq_counts.tsv dataset, *n* = 407). Patients with high E0‐signature gene expression (*CD93* and *ADAMTS1*) showed worse overall survival than those with low expression (*p* < .001), similar to that for patients with high E3‐signature gene expression (*VWF* and *APOD*; *p* < .001; Figure 4F and G).

### 2.5 Inflammatory cancer‐associated fibroblasts (iCAFs) are associated with GC growth and invasion

**FIGURE 5:**
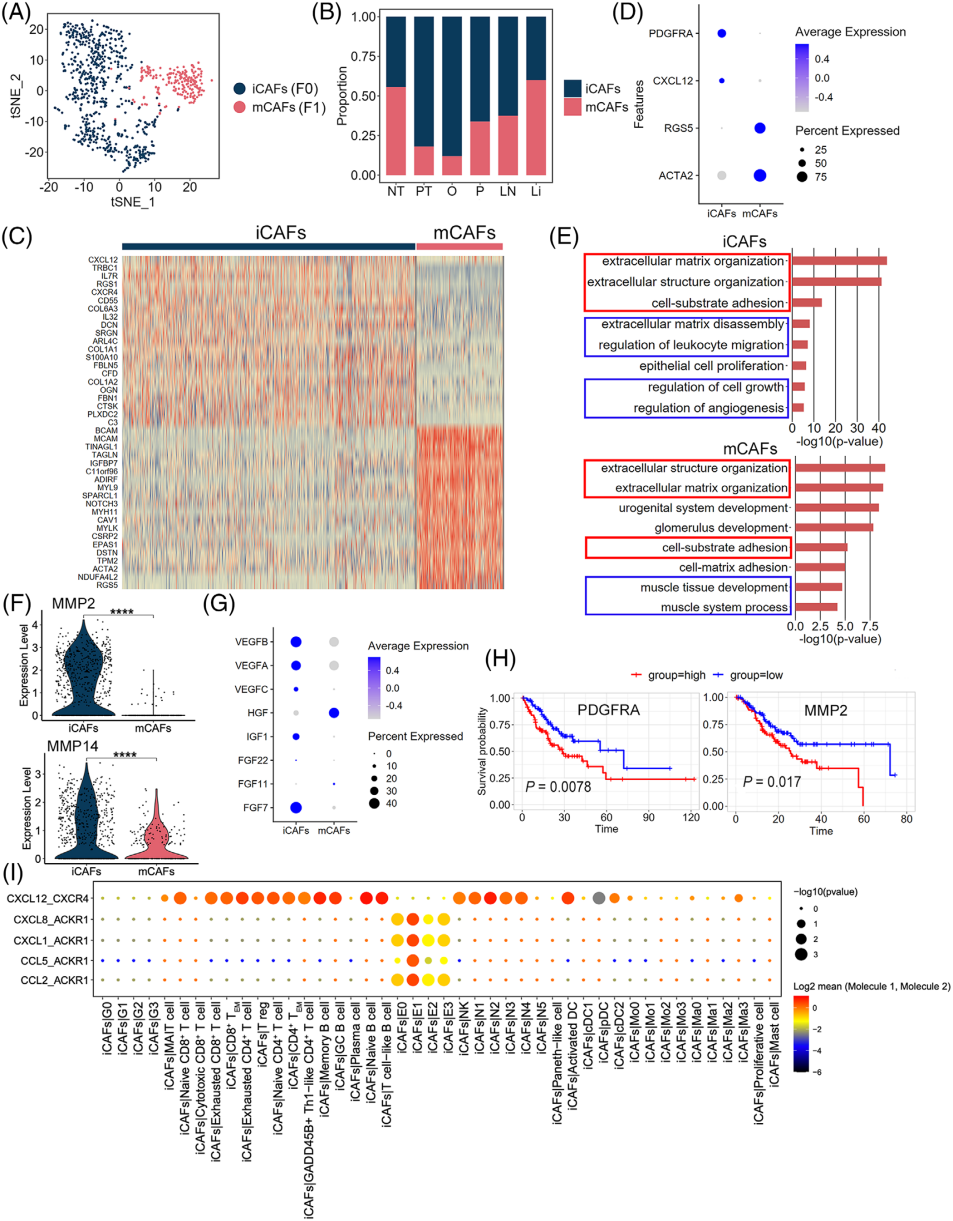
iCAFs could be identified in the TME of GC and are associated with tumour invasion. (A) tSNE of iCAFs (F0) and mCAFs (F1). (B) Relative proportion of iCAFs and mCAFs in NT, PT, O, P, LN, and Li. (C) Heatmap of DEGs between iCAFs and mCAFs. (D) Dot plot showing iCAFs expressing *PDGFRA* and *CXCL12* and mCAFs expressing *RGS5* and *ACTA2*. (E) GO enrichment analysis showing the top enriched functions in iCAFs and mCAFs based on the DEGs. The functions in the red frame mean the common functions between iCAFs and mCAFs; functions in the blue frame represent unique functions of iCAFs or mCAFs. (F) Violin plot showing the expression of *MMP2* and *MMP14* in iCAFs and mCAFs. ^****^ *p* < .0001. (G) Dot plot showing the expression level of growth factors across iCAFs and mCAFs. (H) High levels of iCAF‐associated genes, *PDGFRA* and *MMP2*, predicted poor prognosis in the TCGA‐STAD.htseq_counts.tsv dataset (*n* = 407 patients). Log‐rank *p* < .05 was considered statistically significant. (I) Bubble plots exhibiting significant interactions between iCAFs and other cell groups by ligand–receptor pairs of cytokines

We clustered 889 fibroblast cells into two distinct subclusters after unsupervised cell clustering (Figures 5A–5C, S10A and D). Subcluster F0 was the primary fibroblast type in PT and M and strongly expressed *PDGFRA* and *CXCL12* (Figure 5D), similar to that in the iCAFs described in bladder urothelial carcinoma.^69^ F1 cells highly expressed *RGS5* and *ACTA2* (Figure 5D) and were identified as the myo‐cancer‐associated fibroblasts (mCAFs).^69^ Similar to F0, the proportion of F1 cells was diverse in different samples (Figure 5B). We conducted GO enrichment analysis to explore iCAF and mCAF functions based on the upregulated DEGs between these two types and found that the biological functions extracellular matrix/structure organisation and cell–substrate adhesion were enriched in both types, whereas mCAFs were enriched in the muscle‐related processes and iCAF‐associated terms individually focused on extracellular matrix disassembly, the regulation of leukocyte migration, and the regulation of cell growth and angiogenesis (Figure 5E). *MMP2* and *MMP14*, affecting the top enriched biological functions, were mainly expressed in iCAFs (Figure 5F and Table S8) and can promote extracellular matrix degradation and tumour cell invasion.^70^ *CXCL12* was also a specific marker in iCAFs (Figure 5D), and its high expression in CAFs could promote GC cell invasion and EMT.^68^ To confirm whether iCAFs can regulate cell growth and angiogenesis, the expression of various growth factor‐encoding genes (*VEGF*, *IGF*, and *FGF* families) was analysed in iCAFs and mCAFs; iCAFs expressed more (Figure 5G), suggesting that they can promote GC growth. An analysis of CCI (Figure 5I) showed that the ligand–receptor pair CXCL12‐CXCR4 was involved in the interactions between iCAFs and T, B, or myeloid cells, which is a mechanism of immune resistance in GC.^68^ Another ligand–receptor pair, CXCL1/CXCL8/CCL2/CCL5‐ACKR1 was predicted to act between iCAFs and E1, which might regulate GC tumour progression.^68^ Moreover, patients with GC and higher iCAF signature gene expression showed worse overall survival (*p* < .05) than those with low expression (Figure 5H).

### 2.6 Diversity within the myeloid cell lineage in GC

Subclustering of myeloid cells is shown in Figure S11A; 1801 mononuclear phagocyte system cells [326 dendritic cells (DCs), 894 macrophages and 581 monocytes], 139 mast cells, and 3579 neutrophils were identified by clustering with canonical marker gene expression (Figure S11B and C). The macrophages and monocytes were transcriptionally different in the same sample (Figure S12).

**FIGURE 6:**
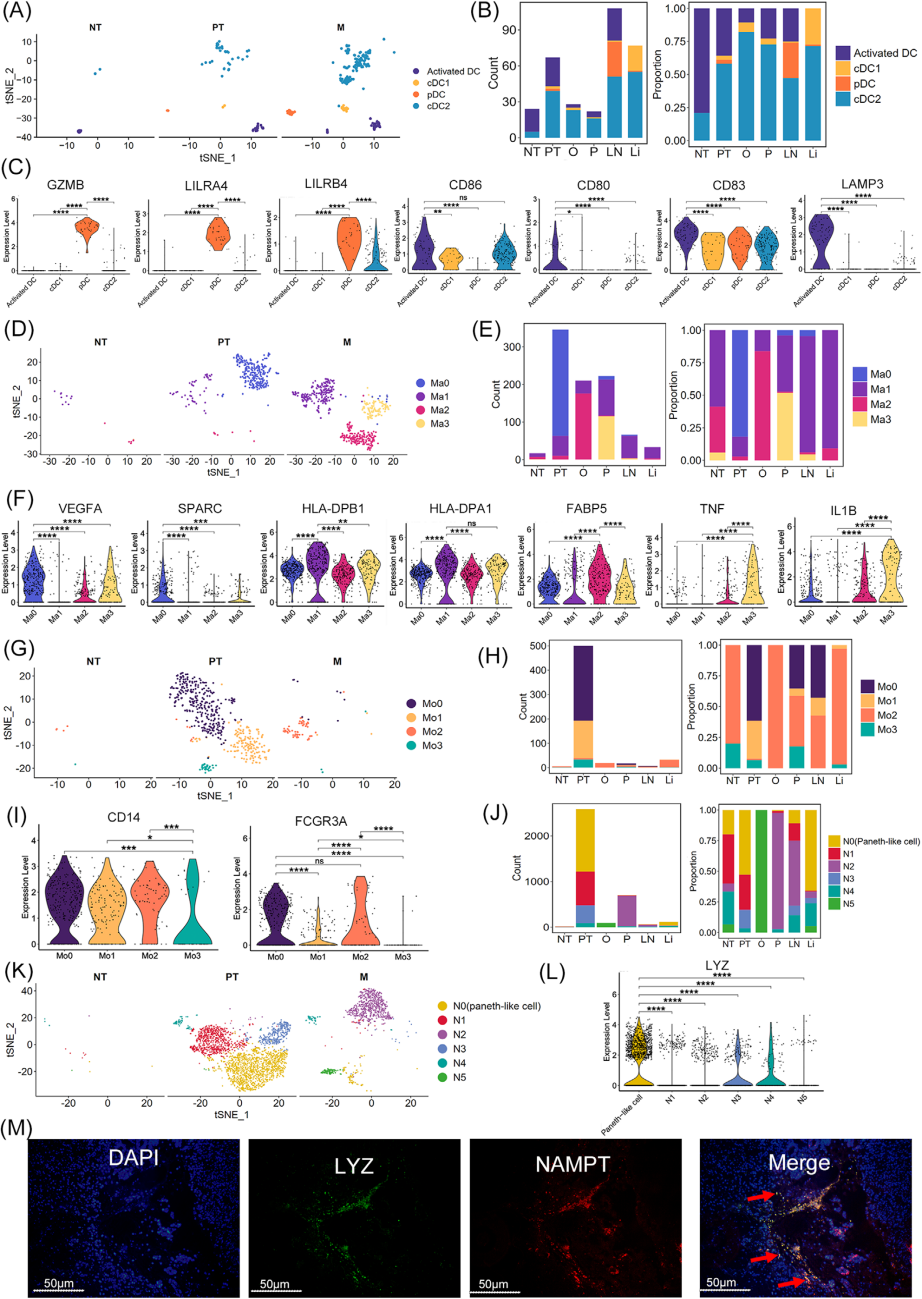
Myeloid cells are abundant during GC progression. (A) tSNE of dendritic cells (DCs) in NT, PT, and M. (B) Counts and relative proportions of each DC cell cluster in NT, PT, O, P, LN, and Li. (C) Violin plots showing the expression of *GZMB*, *LILRA4*, *LILRB4*, *LAMP3*, *CD68*, *CD80*, and *CD83* in each DC cluster. ^*^ *p* < .05; ^**^ *p* < .01; ^***^ *p* < .001; ^****^ *p* < .0001; ns means *p* > .05. (D) tSNE of macrophage cells (Ma) in NT, PT, and M samples. (E) Counts and relative proportions of each macrophage cell cluster in NT, PT, O, P, LN, and Li. (F) Violin plots showing the expression of *VEGFA*, *SPARC*, *HLA‐DPB1*, *HLA‐DPA1*, *FABP5*, *TNF*, and *IL1B* in macrophage clusters (Ma0‐Ma3). ^**^ *p* < .01; ^***^ *p* < .001; ^****^ *p* < .0001; ns means *p* > .05. (G) tSNE of monocyte clusters (Mo0‐Mo3) in NT, PT, and M. (H) Counts and relative proportions of each monocyte cell cluster in NT, PT, O, P, LN, and Li. (I) Violin plots showing the expression of *CD14* and *FCGR3A* (*CD16*) in monocyte clusters. ^*^ *p* < .05; ^***^ *p* < .001; ^****^ *p* < .0001; ns means *p* > .05. (J) Counts and relative proportions of each neutrophil cluster in NT, PT, O, P, LN, and Li. (K) tSNE of neutrophils clusters (N0‐N5) in NT, PT, and M. (L) Violin plots showing the expression of *LYZ* in each neutrophil cluster. ^****^ *p* < .0001. (M) RNAscope staining indicates the co‐expression of LYZ, NAMPT, and DAPI (nuclei) on Paneth‐like cells in PT

DCs were re‐classified into classical DC1 (cDC1), classical DC2 (cDC2), plasmacytoid DCs (pDCs) and activated DCs, with differential proportions among all samples, showing heterogeneity in GC (Figures 6A and S11D). cDC1 and cDC2 were enriched in PT and M, whereas the proportion of activated DCs was large in NT (Figure 6B). Interestingly, pDCs were rarely found in NT but appeared in LN and some PT (Figure 6B). pDCs highly expressed the granzyme gene *GZMB* and leukocyte immunoglobulin‐like receptor family genes *LILRA4* and *LILRB4* and lost expression of *CD86*, *CD80*, *CD83*, and *LAMP3* (Figure 6C), demonstrating an immunosuppressive phenotype.^71^ The results generated from CCI analysis predicted that the ligand–receptor pair HLA‐E‐KLRC1 was also involved in pDCs and NK cells (Figure S11E).

Four subclusters of macrophages (Ma0–Ma3) were transcriptionally heterogeneous (Figure 6D and E). Macrophages are commonly divided into two distinct subsets: M1 and M2.^72^ We investigated the expression of M1 (e.g., *TNF*, *CXCL9*, *CXCL10*, and *IL12A*) and M2 (e.g., *TGFB1*, *CD163*, *CCL18*, and *MRC1*) signature genes in Ma0–Ma3.^73^ The four subclusters of macrophages did not show distinct M1 or M2 signature expression profiles (Figure S13A). Instead, they had similar expression patterns for both M1 and M2 signature genes; thus, we further calculated the M1 and M2 signature scores of Ma0–Ma3 subclusters based on these genes. As shown in Figure S13B, Ma1 and Ma3 subclusters displayed a higher M1 signature score with no obvious tendency in Ma0 and Ma2, indicating that Ma1 and Ma3 cells might be more inclined to have an M1 signature. Subsequently, the biological functions of Ma0–Ma3 were determined via their DEGs. Ma0 cluster was populated in PT and highly expressed *VEGFA* and *SPARC* (Figure 6F), which are essential macrophage genes that induce angiogenesis and cancer cell migration. CCI analysis predicted that E0–E3 cells receive potential angiogenic stimulatory signals from Ma0 through VEGFA and its receptors FLT1/KDR (Figure S11E). Therefore, Ma0 might be an angiogenesis‐associated macrophage type. Ma1 cells were mainly detected in M particularly P, Li, and LN (Figure 6E). Ma1 cells expressed DEGs including *HLA‐DPB1* and *HLA‐DPA1* (MHC‐II) (Figure 6F), which are associated with a pro‐inflammatory phenotype.^74^ The remaining clusters presented origin‐specific heterogeneity and various macrophage characteristics, including *FABP5^+^* Ma2 cells from O and pro‐inflammatory Ma3 cells (*TNF^+^*, *IL1B^+^*)^74^ from P (Figure  6E and F). The *FABP5* expression in Ma2 is closely associated with immunosuppression.^75^ Monocytes were assigned to two known types, *CD14* ^+^ *CD16* (*FCGR3A*)*^−^* classical monocytes (Mo1 and Mo3) and *CD14* ^+^ *CD16* ^+^ intermediate monocytes (Mo0 and Mo2; Figure 6G–I). PT contained large amount of Mo0, Mo1, and Mo3 monocytes. In contrast, M particularly O and Li were enriched in Mo2 cells (Figure 6H). IPA showed that Mo2 cells had increased functions of engulfment and cell death of tumour cells (Figure S11F); thus, Mo2 cells might be responsible for cytotoxic effects toward tumour cells. Subclustering of neutrophils revealed six clusters (Figure 6J and K). One subcluster, N0, was mainly found in PT, and Li and LN expressed high levels of *LYZ* (Figure 6J and L). Lysozyme encoded by *LYZ* is an antimicrobial peptide secreted by Paneth cells which are related to autophagy and the maintenance of immune homeostasis.^76^ Consistently, IPA showed that the function of engulfment and phagocytosis was increased in N0 cells, indicating that they are Paneth‐like cells in GC (Figure S11G). The existence of Paneth‐like cells in GC was further confirmed in the primary tumour sections by RNAscope with the marker genes *LYZ* and *NAMPT* (Figure 6M). Qrigin‐specific heterogeneity and diverse neutrophils characteristics were also detected in the remaining subclusters (Figure S11G). N2 cells, predominantly found in M, especially in P and LN, highly expressed *CXCR4* (Figure S11H), one of the marker genes of pro‐tumoural neutrophils.^77^ It reported that the *CXCR4* ^+^ neutrophils could promote tumour migration and support metastasis.^78^, ^79^ Consistently, IPA showed that the pathways of invasion of tumour cell lines, angiogenesis, and vasculogenesis were strongly activated in N2 cells based on the DEGs between N2 and other neutrophils (Figure S11G). Therefore, N2 cells might be the pro‐tumoural neutrophils with pro‐angiogenic and pro‐invasive properties. N5 cells were mainly found in O (Figure 6J), whereas *PLCG2*, which controls the recruitment and infiltration of neutrophils to the inflammatory microenvironment, was highly expressed in N5 cells (Figure S11H).^80^, ^81^ Functional analysis via IPA showed that cell death of epithelial cells was activated in N5 cells, whereas invasion and migration of tumour cell lines and angiogenesis were inhibited, based on the DEGs between N5 and other neutrophils (Figure S11G). Therefore, N5 cells might be recruited to the ovary metastasis site and inhibit tumour progression. Tumour‐derived granulocyte‐macrophage colony‐stimulating factor (GM‐CSF) can activate neutrophils and induce *PD‐L1* expression, which in turn suppresses T‐cell immunity via its interaction with PD‐1 on T cells and results in GC cell growth.^82^, ^83^ Thus, we investigated the expression of *PD‐L1* (*CD274*) in the neutrophil subclusters. N1 cells highly expressed *PD‐L1*, while other neutrophil subclusters rarely expressed this gene (Figure S14A), indicating that N1 cells might be activated by tumour‐derived GM‐CF. Consistently, CCI analysis exhibited that N1 cells could receive GM‐CSF from G2, G3, E0, and MAIT cells through the ligand–receptor pair of CSF2 (i.e., GM‐CSF) and CSF2RA/CSF2RB/CSF1R/CSF3R (Figure S14B). Additionally, the ligand–receptor pair PD‐L1/PD‐1 was involved in the interactions between N1 and T‐cell clusters (i.e., exhausted CD8^+^ T, cytotoxic CD8^+^ T, exhausted CD4^+^ T, CD8^+^ T_EM_, CD4^+^ T_EM_, Treg, and MAIT cells; Figure S14C), indicating that N1 cells might suppress T‐cell immunity through PD‐L1/PD‐1 interactions. CCI analysis also predicted macrophage subclusters, monocyte subclusters, and neutrophils subclusters interacted with cytotoxic/exhausted CD8^+^ T cells and/or NK cells through the ligand–receptor pair HLA‐E‐KLRC1/KLRC2 (Figure S11I). Due to the immunosuppression and pro‐angiogenesis functions of tumour‐associated macrophage, we performed an in vitro experiment by isolating the macrophages (monocyte‐derived) and NK cells from human peripheral blood mononuclear cells and co‐culturing them to investigate the role of HLA‐E‐KLRC1 signalling (detailed methods were shown in the Supplementary Materials). As a result, the amount of interferon (IFN)‐γ was significantly improved following anti‐NKG2A antibody treatment compared with the isotype (Figure S15), indicating that targeting the HLA‐E‐KLRC1 signalling might be a novel clinical therapeutic opportunity in GC.

Myeloid‐derived suppressor cells (MDSCs) represent a heterogeneous population of immature myeloid cells including monocytic MDSCs (M‐MDSCs), granulocytic or polymorphonuclear MDSCs (G‐MDSCs), and immature or early‐stage MDSCs (e‐MDSCs), which have immune suppression properties in cancer.^84^, ^85^, ^86^ M‐MDSCs are morphologically and phenotypically similar to monocytes and are identified as CD33^+^CD11b^+^CD14^+^HLA‐DR^–/low^ in humans. Meanwhile, G‐MDSCs are morphologically similar to neutrophils and are defined as CD33^+^CD11b^+^CD15^+^ Lox‐1^+^CD14^–^HLA‐DR^–/low^, and e‐MDSCs are defined as CD33^+^CD11b^+^HLA‐DR^–^ CD14^–^ CD15^–^.^84^, ^85^, ^86^ As MDSCs can be identified as distinct clusters within the lineages of neutrophils, monocytes, macrophages, and DCs,^87^, ^88^, ^89^, ^90^ we rechecked these lineages using the marker genes of MDSCs. As shown in Figure S16A, Ma2 was identified as M‐MDSCs. Moreover, functional analysis using IPA, based on the DEGs that were identified between Ma2 and other macrophages, revealed that the Ma2 cells activated the T‐cell exhaustion, inducible nitric oxide synthase (iNOS), and VEGF signalling pathways, as well as increased the production of nitric oxide (NO) and reactive oxygen species (Figure S16B). This was in line with the characteristics and immunosuppressive functions of M‐MDSCs.^84^, ^85^ Therefore, Ma2 cells might be the M‐MDSCs that induced immunosuppressive activity in GC. Moreover, *OLR1* (LOX‐1) is highly associated with G‐MDSCs at the tumour site, whereas G‐MDSCs has a unique gene expression profile compared with neutrophils.^91^ Based on the expression of *OLR1* (LOX‐1), only N0 (Paneth‐like cells) and N3 clusters were defined as *OLR1* ^+^ neutrophils (Figure S16C). Meanwhile, IPA showed that engulfment and phagocytosis was positively regulated in N0 cells (Paneth‐like cells; Figure S11G). Since G‐MDSCs have lower phagocytic activity,^92^ the N0 cells (Paneth‐like cells) may not be G‐MDSCs in our GC samples. In N3 cells, IPA revealed that the pathways of iNOS signalling, IL‐6 signalling, and oxidative phosphorylation were activated (Figure S16D), whereas migration of tumour cell lines and epithelial–mesenchymal transition were positively regulated, and the phagocytosis of cells and cytotoxicity of lymphocytes were inhibited (Figure S16D and E). These findings were consistent with the characteristics and immunosuppressive functions of G‐MDSCs.^93^, ^94^ Therefore, N3 cells may indeed be G‐MDSCs in our GC samples.

### 2.7 T and B lymphocyte subclusters vary among different organ metastases and a 20 gene signature of lymph node‐derived exhausted CD8^+^ T cells was validated to predict lymph node metastasis

**FIGURE 7:**
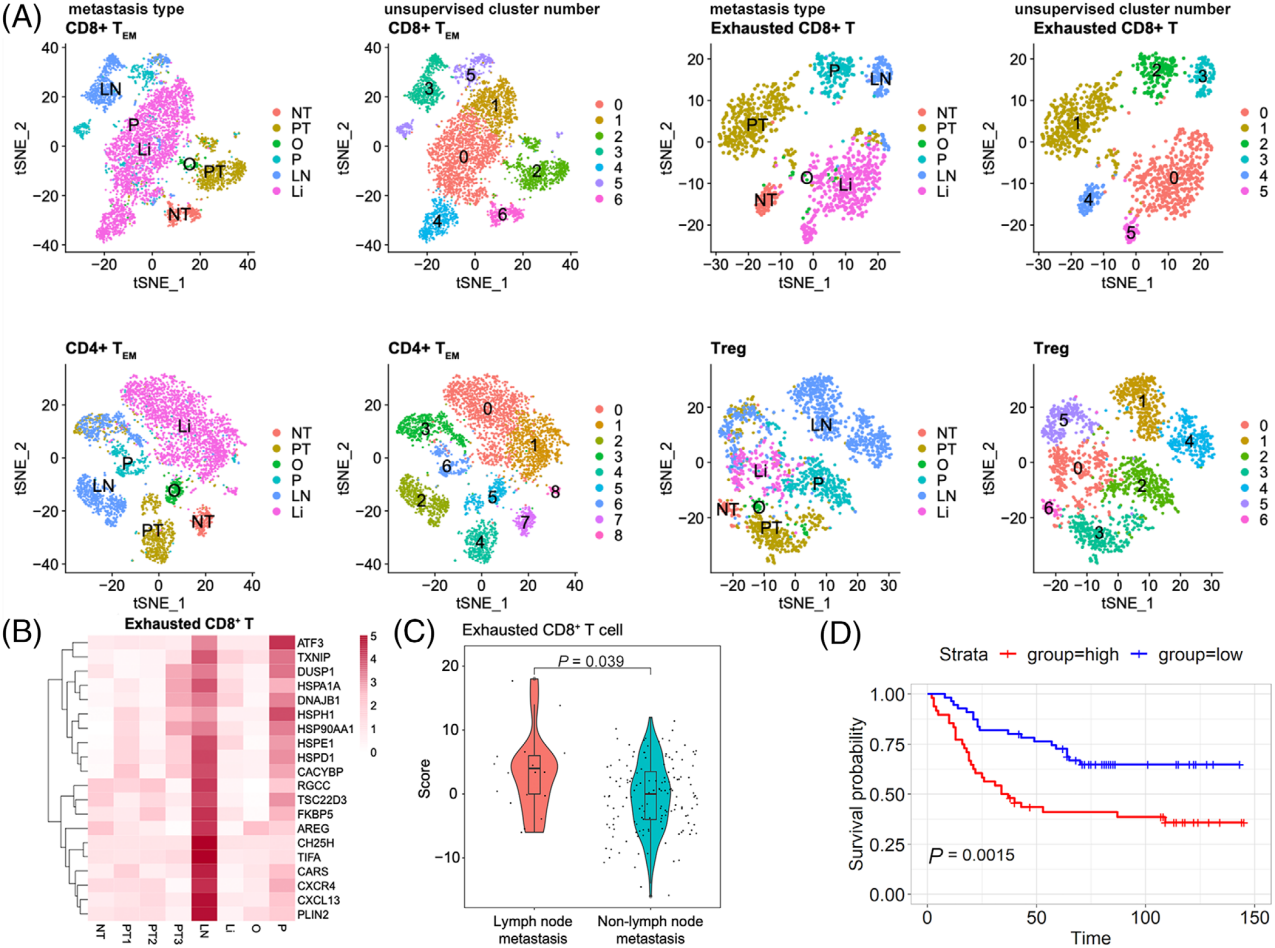
T and B lymphocyte subclusters vary among different organ metastases. (A) tSNE of CD8^+^ T_EM_, exhausted CD8^+^ T, CD8^+^ T_EM_, and Tregs coloured with metastasis type and unsupervised cluster number. (B) Heatmap of top 20 DEGs in exhausted CD8^+^ T cells in the different samples. (C) The violin plot shows a significant difference in the mean score of the 20‐gene signature from lymph node‐derived exhausted CD8^+^ T cells between the non‐lymph node metastasis (*n* = 17 intestinal‐histology and mixed‐histology patients) and lymph node metastasis (*n* = 146 intestinal‐histology and mixed‐histology patients) groups in the primary GC dataset (GSE62254). The box plots represent the median, bottom and top quantiles, whiskers correspond to 1.5× the interquartile range. Log‐rank *p* < .05 was considered statistically significant. (D) Prognostic significance of the 20‐gene signature in exhausted CD8^+^ T cells derived from the lymph node metastasis samples was validated in the GC dataset (GSE84437, *n* = 433 patients). Log‐rank *p* < .05 was considered statistically significant

Tumour‐infiltrating T or B lymphocytes play critical roles in cancer development and metastasis^95^; thus understanding the infiltrating status of these immune cells in the GC metastases of different organs might develop specific diagnostic and therapeutic strategies for organ‐specific metastases. Herein, each subpopulation of T or B lymphocytes was re‐clustered with unsupervised method. The resulting data were coloured with unsupervised cluster number or metastasis type to explore organ‐specific metastatic features of lymphocytes in GC. As shown in the Figures 7A and S17A, different organ metastases were transcriptionally heterogeneous in most T subclusters and some B subclusters as they were clearly separated from each other in t stochastic neighbour embedding (tSNE) plots. For instance, in exhausted CD8^+^ T cells, different organ metastases‐derived cells were clustered in different subsets. Similar trends were found in CD8^+^ T_EM_, CD4^+^ T_EM_, Tregs, GADD45B^+^ Th1‐like CD4^+^ T cells, naïve CD4^+^ T cells, switch memory B cells, and T cell‐like B cells, suggesting that these subclusters displayed different phenotypes when present in different organ metastases in GC.

The incidence of lymph node metastasis in early GC is extremely high with the percentage of 8.7–24.6% and the lymph node status has been found to be closely related to disease prognosis^96^; thus it is of great significance to identify the signature of lymph node metastasis at the single‐cell resolution. Therefore, we sought to generate gene expression signatures of lymph node‐derived T or B subclusters in GC. We performed single‐cell DEGs analysis on each T or B subpopulation between lymph node‐derived and non‐lymph node‐derived subsets, followed by selecting top 20 upregulated DEGs of lymph node‐derived subset. After this process, the 20‐gene signature in each lymph node‐derived T or B subcluster was obtained. As shown in Figures 7B and S17B, in exhausted CD8^+^ T cells, expression of the top 20 upregulated DEGs of the lymph node‐derived subset was higher in LN than in other metastases, which were also higher expressed in PT3 (the primary tumour sample from the patient who had lymph node metastasis) compared with other primary tumour, suggesting the 20‐gene signature reflects the patterns of metastatic spread from the primary site to lymph node. A consistent trend was also observed in the CD8^+^ T_EM_, CD4^+^ T_EM_, Tregs, and exhausted CD4^+^ T cells, as the expression of the top 20 upregulated DEGs of each subset was high in LN and PT3, but was low in the other T or B subpopulations. Then, the 20‐gene signatures of CD8^+^ T_EM_, exhausted CD8^+^ T cells, Tregs, CD4^+^ T_EM_, and exhausted CD4^+^ T cells were validated in an independent GC cohort (GSE62254). For each tumour sample in these data, a score of 20‐gene signature from lymph node‐derived T subpopulation was calculated using bulk RNA‐seq data with a similar method described previously,^13^ and sample was categorised into either the primary GC with lymph node metastasis or the primary GC without lymph node metastasis (including other type metastases and the primary tumour without any metastases) group. In Figure 7C, only the score of the 20‐gene signature from lymph node‐derived exhausted CD8^+^ T cells was significantly higher in the primary GC with lymph node metastasis compared with that in the non‐lymph node metastasis set (*p* < .05). Furthermore, the prognostic significance of this 20‐gene signature in exhausted CD8^+^ T cells derived from the lymph node metastasis samples was evaluated in a large‐scale GC cohort (GSE84437). The results showed that patients with high scores of this 20‐gene signature in exhausted CD8^+^ T cells derived from the lymph node metastasis samples, had a significantly shorter survival than those with lower scores (*p* < .01) (Figure 7D), indicating great potential of this 20‐gene signature to prognosticate patient survival.

## 3 DISCUSSION

Intra‐ and inter‐tumour heterogeneity in the primary tumour and different metastases limit the effects of chemotherapy and immunotherapy on GC, thereby hindering treatment and outcome prediction. Therefore, a comprehensive understanding of the biological heterogeneity in primary GC and metastases is urgently needed. Herein, using scRNA‐seq, both heterogeneity and core gene expression signatures for specific carcinoma cell subtypes were illustrated, and a distinct and dynamic TME was identified in the primary tumour and different metastases.

We have identified a high degree of carcinoma cell heterogeneity during GC progression by identifying diverse malignant epithelium subsets and their distinct roles. Notably, a rare cell type, G3, with strong EMT and stemness signatures was identified in PT and different M. Mounting evidence suggests that EMT induction strongly affects tumour progression, facilitating cancer cell invasion and metastasis to distant sites generating stem‐like cells, which is associated with drug resistance in cancer.^97^ Consistently, clinical data showed that Patient 3 (O1), with a higher proportion of G3 cells, exhibited resistance to the PD‐1 inhibitor and paclitaxel‐albumin combined therapy. Moreover, G1 cells exhibited EMT characteristics with peritoneal dissemination and were only found in the ovary and peritoneal metastases. A high frequency of ovarian metastasis with peritoneal dissemination has previously been reported,^98^ indicating that G1 cells are a characteristic of these metastases. Two branches, invasive and angiogenic G0 and dormancy‐like G2, appeared in the trajectory graph reflecting different states of malignant epithelial cells. Dormant cancer cells have been investigated in primary tumours and metastases like in breast cancer and GC.^99^, ^100^ These cells enter dormancy, evade therapies and are highly associated with local recurrence or metastasis, which has become a new challenging area in cancer therapy.^100^ We found that high expression of G0‐, G1‐ or G3‐associated genes can predict poor survival for patients with GC, suggesting that these three subclusters are more aggressive and that these core gene signatures could serve as biomarkers of prognosis. We used CopyKAT to detect CNVs in malignant epithelial cells in GC primary tumour sites and metastases. Most of the metastases shared similar subclone 1 except one liver metastasis from Patient 1 who had distal gastric adenocarcinoma with liver metastasis. Moreover, CNVs in malignant epithelial cells showed inter‐ and intra‐tumour heterogeneity of subclones between the GC primary tumour sites and metastases.

An immunosuppressive characteristic was observed in MAIT in M and T cell‐like B cells. In contrast to previous studies showing that MAIT cells kill tumour cells, recent studies reported that MAIT cells exhibit tumour‐promoting effects by suppressing T and/or NK cells in lung cancer,^101^ and MAIT cells with an exhausted phenotype would indicate poor outcome in hepatocellular carcinoma.^102^ Consistently, MAIT cells in M presented a diminished effector‐memory T‐cell phenotype with no cytotoxic responses compared with those in PT and NT. Meanwhile, a higher proportion of T cell‐like B cells was identified in M, which was in line with a previous finding that the percentage of T cell‐like B cells in the peripheral blood of patients with GC metastases is significantly higher than those in patients without metastases,^103^ indicating that T cell‐like B cells might play some roles in GC metastasis. This study suggested that T cell‐like B cells are associated with the inhibition of T and NK cell effects and the binding of endothelial cells. Although more functional assays are warranted, MAIT and T cell‐like B cells might provide a new approach for GC anti‐tumour therapy. We also found that *PD‐1* expression in CD8^+^ T cells might predict the clinical response to PD‐1 blockade in GC, which is consistent with that in other cancer types like hepatocellular carcinoma.^104^ More importantly, based on the clinical data (Patients 3 and 6), the expression of *PD‐1* in CD8^+^ T cells could be more predictive and suitable than the CPS for identifying GC patients who will respond effectively to anti‐PD‐1 therapy. Additionally, previous studies have indicated that the simultaneous blockade of multiple inhibitory receptors leads to the synergistic reversal of T‐cell dysfunction and improves the disease control ratio in cancer.^105^, ^106^ Based on the high expression of several inhibitory receptors (Figures 3F and S8), combinatorial therapy targeting specific co‐inhibitory receptors may exert beneficial clinical effects for patients 1, 2, 5, and 6.

A heterogeneous cellular milieu with active crosstalk between stromal cells and other cell clusters was also highlighted. E0 and E3 cells can receive the potential angiogenic signals, VEGFA, from G0 and Ma0 cells via VEGFR1, illustrating a pro‐metastatic microenvironment for GC progress. More importantly, both E1 cells and iCAFs were found to acquire immunosuppressive properties related to *CXCR4* ^+^ immune cell recruitment into the stroma. Therefore, CXCR4 antagonists such as AMD3100 which is frequently used in GC to block the CXCL12/CXCR4 axis might be effective against E1 cells and iCAFs.^68^

Furthermore, Ma2 cells, which were found in GC primary tumours and sites of metastases, were identified as M‐MDSCs. Recently, MDSCs have been detected in various cancers and are a promising target for cancer therapy, including melanomas, as well as lung and breast cancers.^85^ The main functions of MDSCs are to escape immune surveillance and affect tumour angiogenesis and TME to promote tumour progression.^85^, ^107^ M‐MDSCs often upregulate NO and produce immunosuppressive cytokines, such as TGF‐β, to suppress T cells,^84^, ^85^, ^108^ which were also observed in this study. Therefore, targeting MDSCs might be a novel approach for GC treatment. Notably, one neutrophil cluster, Paneth‐like cells, was found in GC primary tumours and metastases. Although Paneth‐type granules have been reported in lymphatic and intraperitoneal metastasis in synchronous cancers of the oesophagus and ampulla of Vater and in primary GC,^109^, ^110^ this is the first report of their existence in lymph node and liver metastasis of GC. Although the detailed roles of this cluster remain unclear, their function could be related to engulfment and phagocytosis (Figure S11G). Another subcluster of N1 was activated by tumour‐derived GM‐CSF, which then suppressed T‐cell immunity through the PD‐L1/PD‐1 interaction. This is consistent with previous research that demonstrated PD‐L1 expression on tumour‐activated neutrophils in GC,^82^ and provides a potential strategy to overcome immune suppression in GC. Additionally, N3 cells were identified as G‐MDSCs, showing immunosuppressive and tumour‐promoting properties. Thus, G‐MDSC may be targeted for GC therapy in the future.

Importantly, HLA‐E‐KLRC1/KLRC2 signalling was predicted to function between many cell subsets in the TME. Normally, tumour cells expressing HLA‐E activate the KLRC1 receptor in cytotoxic lymphocytes and protect themselves from lysis.^53^ In this study, not only the cancer subclusters (G0, G1, and G3) but the E1, MAIT in M, T cell‐like B cells, pDCs, Ma0–Ma3, Mo0–Mo3, and N0–N5 were predicted to express HLA‐E binding KLRC1/KLRC2 in cytotoxic/exhausted CD8^+^ T cells and/or NK cells, which might induce immunosuppression to promote GC metastasis. Consistently, a previous study showed that epithelial cancer cells, as well as tumour DCs and macrophages, contributed to HLA‐E enrichment in adenocarcinoma including esophageal, colorectal, lung, and kidney cancer, and this enrichment related to KLRC1 upregulation on tumour‐infiltrating CD8^+^ T lymphocytes.^111^ In a previous study, which isolated mixtures from GC patient tumour tissue samples with at least 5% of NKG2A^+^ (KLRC1) tumour‐infiltrating T lymphocytes (TILs), HLA‐E‐KLRC1 interaction negatively affected IL2 receptor–dependent CD8^+^ TIL proliferation and contributed to CD8^+^ TIL dysfunciton.^111^ Another study showed that when human colorectal tumour spheroids from colorectal tumour patients were co‐cultured with CD8^+^ T and NK cells, the expression of HLA‐E was strongly induced, with tumour cells evading the immune response through HLA‐E‐KLRC1.^112^ Besides, haematological malignant cells co‐cultured with NK cells under cytomegalovirus infection increased NKG2C^+^ (KLRC2) NK cell expansion and enhanced their anti‐tumour cytotoxicity.^113^ The present study found that the amount of IFNγ was significantly improved following anti‐NKG2A antibody treatment compared with the isotype (Figure S15) when co‐culture of macrophages and NK cells. Anti‐KLRC1 (NKG2A) monoclonal antibody (Monalizumab) has been proposed as a novel checkpoint inhibitor used in clinical trials to treat several carcinomas.^56^ Currently, there is no such antibody used to treat GC patients in clinical trial, and thus using anti‐KLRC1 (NKG2A) monoclonal antibody in GC is a potentially effective treatment strategy.

More intriguingly, a 20‐gene signature of lymph node‐derived exhausted CD8^+^ T cells was discovered and validated to predict lymph node metastasis in GC. Although evidence has shown that exhausted CD8^+^ T cells closely related to lymph node metastasis in breast cancer and melanoma,^114^ this study was the first time to use the gene signature of lymph node‐derived exhausted CD8^+^ T subcluster to forecast lymph node metastasis in GC. In the top 20 upregulated DEGs of lymph node‐derived exhausted CD8^+^ T cells (Figure 7B), *CXCL13*, *CXCR4*, *CH25H*, *HSPD1*, *HSP90AA1*, and *ATF3* have been confirmed to promote lymph node metastasis in several cancer types.^115^, ^116^, ^117^, ^118^, ^119^, ^120^ Especially, previous studies uncovered that the serum expression level of *CXCL13* in GC patients with lymph node metastasis was significantly higher than that in those without lymph node metastasis^115^ and overexpression of *CXCR4* in primary gastric cancers was certified as an independent risk factor for lymph node metastasis,^116^ while *HSP90AA1* was correlated with *MMP9* to promote cell invasion and lymph node metastasis in GC patients.^119^ Thus, these data further support our findings and highlight the clinical value of the 20‐gene signature in exhausted CD8^+^ T cells derived from the lymph node metastasis samples to diagnose and prognose lymph node metastasis of GC.

In summary, this is the first study to provide insight into the intra‐ and inter‐tumour heterogeneity of GC primary tumour and different organ metastases at a single‐cell resolution level, illustrating correspondence between cancer cells and TME. We defined the population structure and cellular status underlying GC progression and correlated several marker genes of distinct subsets to clinical outcomes in humans. The functional relevance of distinct subclusters for GC progression was also revealed. Since there are various methods to detect somatic mutations and distinct subclones from scRNA‐seq data,^121^, ^122^, ^123^ it is accessible and worthwhile to perform the parallel profiling of the genetic and transcriptional heterogeneity of this GC data in the future, which will provide valuable information regarding the somatic mutation evolution and functionality in primary and metastatic GC. Although the sample size of scRNA‐seq analysis was small, our findings were validated in several bulk RNA‐seq cohorts with more than 1100 patients. Our study provides insight into GC biology and will promote personalised medicine for this disease.

## 4 METHODS

### 4.1 Human specimens for scRNA‐seq analysis

All procedures performed in studies involving human participants were in accordance with the ethical standards of the Institutional Review Board at First Affiliated Hospital, Zhejiang University School of Medicine (No. 2018–309) and with the Helsinki Declaration (as revised in 2013). Written informed consent was obtained from the patients. In this study, patients diagnosed with GC were enrolled and 10 samples from six patients were totally collected (Table S1). Histological types were determined using the pathological results. Freshly resected biopsies were divided into two parts. One part was processed for the scRNA‐seq experiment, and the other part was used for pathological and histological assessments. All the fresh samples were preserved in Tissue Storage Solution (Miltenyi Biotech, Germany) and processed within 48 h.

### 4.2 Sample dissociation

Briefly, tissues were washed twice using PBS supplied with 10% BSA (Sigma, USA), minced into small pieces with the size of 2–4 mm^3^, and then dissociated using a Human Tumor Dissociation kit (Miltenyi Biotech, Germany). The only exception was the liver biopsy sample which was dissociated with the Mouse Liver Dissociation kit (Miltenyi Biotech, Germany) based on the cell viability results of preliminary experiment. The cell suspension was further filtered through 70 μm SmarterStrainers (Miltenyi Biotech, Germany). Red blood cells were removed using a Red Blood Cell Lysis Solution (10×) (Miltenyi Biotech, Germany). Dead cells were eliminated using a Dead Cell Removal Kit (Miltenyi Biotech, Germany) to increase the efficiency of sorting robust, and the live cells were washed twice, re‐suspended and then used for single‐cell experiments.

### 4.3 Library preparation and single‐cell RNA sequencing

Library preparation was performed according to the Chromium Next GEM Single Cell 3ʹ Reagent Kits v3 (10x Genomics, USA). The libraries were then pooled and sequenced on a Novaseq6000 (Illumina, USA).

### 4.4 Single‐cell RNA expression quantification, quality control and cluster analysis

Raw sequencing data from 10x Genomics were aligned and quantified using the CellRanger (10x Genomics) suite (version 3.0.2). The human genome GRCh38 was used as the reference genome and the CellRanger count module was used to map reads. Raw gene expression matrices for each experimental condition were imported in R software (version 3.6.3) using Seurat R package (version 3.1.5).^124^ We excluded cells with the following criteria: less than 200 unique genes expressed, more than 5000 unique genes expressed or more than 20% of reads mapping to mitochondria. The gene expression matrices of the remaining 42 968 cells were normalized through a global‐scaling method with a default scale factor and natural‐log transformed using log(1+x). For further downstream analysis, variably expressed genes were selected using the FindVariableGenes function in Seurat. Then we combined all samples by the function of IntegrateData and the integrated data was scaled through the function of ScaleData, aiming to remove the unwanted sources of variation. We performed cell cycle scoring and regression to assess the effects of cell cycle heterogeneity on our data analysis, based on the online method of Seurat (https://satijalab.org/seurat/articles/cell_cycle_vignette.html). Each cell was assigned a cell cycle score calculated by the function of CellCycleScoring() based on its expression of G2/M and S phase markers. Then, each cell was allocated different phases (G2/M, S, or G1) according to its cell cycle score. While scaling the data, cell cycle scores were set as variables to regress out and were individually regressed against each feature using a linear model to remove cell cycle effects. The resulting residuals of the model were scaled and centred; cell cycle heterogeneity did not contribute to the PCA results, since the cell clustering was not separated by cell cycle phases with or without cell cycle regression (Figure S18A and B). More importantly, the cell clustering was almost the same between the data with and without cell cycle regression (Figure S18C), indicating that cell cycle heterogeneity had few effects on the analysis. Therefore, we classified cell types without cell cycle effects removal. Then, we selected the most significant 20 principal components, via principal component analysis and used them to perform tSNE dimensionality reduction. Cell clustering was performed using the ‘FindClusters’ function in Seurat, and the clusters were annotated by the expression of canonical marker genes.

### 4.5 Division of non‐malignant and malignant epithelial cells

The bulk RNA‐seq data of stomach adenocarcinoma (dataset ID: TCGA‐STAD.htseq_counts.tsv) were downloaded from TCGA database and adopted to recognise non‐malignant cells and malignant epithelial cells. The top 50 DEGs (adjusted *p* value < .01) in tumour tissues in TCGA data were selected to calculate malignant scores, whereas top 50 DEGs (adjusted *p* value < .01) in normal tissues were selected to calculate non‐malignant scores, using ‘AddModuleScore’ function in Seurat R package (Table S5).^10^ Using the malignant and non‐malignant scores,^10^ the initial putative malignant and non‐malignant epithelial cells were defined. Specifically, malignant score minus non‐malignant score in each epithelial cell was calculated, and the score was ranked from small to large to fit the growth curve. Then, cells were divided as malignant with higher score (> ‐0.02) or non‐malignant with lower score (< ‐0.02) based on the largest gap near the inflection point of the growth curve. The bias in the initial step was due to non‐epithelial cells retained in TCGA tissues. Therefore, in the following steps, the malignant/non‐malignant scores of each epithelial cell were re‐calculated, and the top 50 highly expressed genes (adjusted *p* value < .01) in putative malignant epithelial cells were selected to calculate malignant scores, whereas the top 50 DEGs (adjusted *p* value < .01) in putative non‐malignant epithelial cells were selected to calculate non‐malignant scores. The recognition process was repeated as described earlier until the classification result was consistent.

### 4.6 Inference of developmental trajectory

The cell state transitions were analysed using the Monocle2 algorithm.^125^ The function of ‘newCellDataSet’ with the parameter ‘expressionFamily’ of ‘negbinomial.size’ was used to create a CellDataSet object. The function of dispersion table was performed to determine genes expression, and genes which were detected in less than 10 cells and had a lower expression than 0.001 were filtered out. After dimension reduction, the highly variable genes selected using the FindVariableFeatures function in Seurat with the method of ‘vst’ were provided to Monocle to infer the cell trajectory.

### 4.7 CopyKat performed in malignant epithelial cells

We applied CopyKAT to malignant cells with default parameters and extracted inferred copy number profiles of genes per cell.^35^ The matrix of copy number alteration results was passed to ‘Seurat’ R package to create a new assay for subsequent analysis. After data processing and dimension reduction, all cells were clustered into subpopulations based on the inferred copy number profiles; the function of FindAllMarkers() was used to identify genomic regions with copy number differences of each subpopulation. To annotate the biological function of subpopulations, we performed GO analysis on DEGs with Metascape (http://metascape.org).

### 4.8 Calculate the scores of 20‐gene signatures from lymph node‐derived subpopulations

Each subpopulation of T and B cells was re‐clustered with the K‐nearest neighbour graph and Louvain algorithm. For each subpopulation, if more than 90% cells in a cluster came from LN, we defined the cluster as lymph node‐derived subcluster and the top 20 upregulated DEGs of this cluster were chosen for the score calculation. The expression matrix was extracted from normalised bulk matrix of large‐scale primary GC (GSE62254) and the samples of intestinal type and mix type were reserved. For each sample in this dataset, score of 1 or –1 was assigned for each of the top 20 upregulated DEGs of lymph node‐derived subpopulation based on its relative expression (> median value, the score was assigned 1; ≤ median value, the score was assigned –1).^13^ After that, the scores of each sample were summed, which constituted the signature score.

### 4.9 Survival analysis

RNA‐seq and clinical data of stomach adenocarcinoma patients (cancer study id: stad tcga) were obtained from TCGA using cgdsr R package. The tumour samples were divided into two groups along with low (25%) and high (75%) target gene expression for all patients. Survival analysis was performed using the Kaplan–Meier formula in R package ‘Survival’, and finally, the survival curve was visualised using the ggsurvplot function of the R package ‘survminer’. We also download another large‐scale primary GAC dataset (GSE84437) and divided all samples into two groups along with low (20%) and high (80%) score of 20‐gene signature from lymph node‐derived CD8^+^ T cells. Survival analysis and visualisation were implemented through the R package.

### 4.10 Cell–cell communications analysis

CellPhoneDB^126^ was used to analyse cell–cell communications. The genes that expressed in less than 10 cells were filtered and the filtered gene expression matrix was imported into CellphoneDB (version 2.1.4). We used permutation tests to provide the significance and used default on method parameters. After the results of cell–cell communications were obtained, the ligand–receptor pairs, with no significant mean among all pairs of cell subsets, were filtered out.

CellTalkDB v1.0 was also used to identify significantly enriched ligand–receptor pairs and infer cell–cell communications.^127^ The tool is based on a comprehensive database of ligand–receptor interaction pairs that have been identified in both humans and mice. The interaction pairs are then manually verified using the known protein–protein interactions that are available on the STRING database. Using CellTalkDB v1.0, the proteins that were expressed in more than five cells were selected and the threshold of enriched ligand–receptor pair score was set to 0.1.

### 4.11 IPA

The FindAllMarkers function in Seurat was used to calculate the DEGs list of each cell subset with the following settings: min.pct = 0.2, only.pos = FALSE, min.pct = 0.2 and logfc.threshold = 0.2. The DEGs of each cell subset were obtained by comparing the cell subset with other cells and they were filtered with a cutoff of the adjusted *p* value < .05 (Wilcoxon test). The canonical pathways and diseases and bio functions of the filtered DEGs were analysed using IPA (QIAGEN Inc., https://digitalinsights.qiagen.com/products‐overview/discovery‐insights‐portfolio/analysis‐and‐visualization/qiagen‐ipa/).

### 4.12 GO enrichment analysis

The top 100 upregulated DEGs of each cluster were then used to perform GO analysis using clusterProfiler R package,^128^ and the functional gene sets belonging to biological process were focused on this study. The q value was used to select the significantly enriched results with a cutoff value of .05. The results of GO enrichment analysis are filtered according to the *q* value (qvalueCutoff = .05).

### 4.13 Scoring the proliferative and migrative capacities of G0–G3 cells

The proliferation scores of G0–G3 cells were generated according to the expression of signature genes that represent tumour proliferation ability (*MKI67*, *IGF1*, *ITGB2*, *PDGFC*, *JAG1*, and *PHGDH*),^31^, ^32^ while the migration scores were determined by measuring the expression of signature genes that indicate tumour migration ability (*VIM*, *SNAI1*, *MMP9*, *AREG*, *ARID5B*, and *FAT1*).^21^, ^22^, ^33^, ^34^ Both functional scores were defined as the average normalised expression of the corresponding genes based on previous literature.^129^

### 4.14 Scoring M1 and M2 signatures of Ma0–Ma3 cells

The M1 or M2 signature score of Ma0–Ma3 cells were generated according to the expression of M1 (e.g. *TNF*, *CXCL9*, *CXCL10*, and *IL12A*) or M2 (e.g. *TGFB1*, *CD163*, *CCL18*, and *MRC1*) signature genes.^41^ Both signature scores were defined as the average normalised expression of corresponding genes based on previous literature.^129^

### 4.15 Calculating the correlation coefficient between NT1 and the three GC normal tissue samples

Cell Ranger outputs of three GC normal tissue samples previously reported^6^ were downloaded from https://dna‐discovery.stanford.edu/ and transmitted to the Seurat R package. Three GC normal tissue samples were filtered for the parameters thresholds in the article and combined into one large sample, named ‘CCR’. The top 20 DEGs between normal (CCR and NT1) and tumour (O1, PT1, PT2, PT3, Li1, Li2, LN1, LN2, and P1) samples were identified using FindAllMarkers() for correlation analysis between samples using spearman coefficient based on the average expression of the selected genes. The sum of counts across genes was used to perform correlation analysis between CCR and NT1. Sums were transformed using log(1+x), and Pearson's correlation coefficient was used to calculate the correlation between the two samples.

### 4.16 Multicolor RNA−in situ hybridisation (RNAscope)

Briefly, bake slides of patient tissues were obtained from The First Affiliated Hospital, Zhejiang University School of Medicine. The slides were pretreated using the RNAscope^®^ target retrieval reagents (322000), RNAscope^®^ H202 and protease reagents (PN 322381) (Advanced Cell Diagnostics, USA). Then, they were incubated with the probes targeting *NAMPT* (599311, Advanced Cell Diagnostics, USA), *LYZ* (421441, Advanced Cell Diagnostics, USA), positive control probe‐Hs (320861, Advanced Cell Diagnostics, USA), and negative control probe (320871, Advanced Cell Diagnostics, USA). The RNAscope^®^ multiplex fluorescent v2 detection kit (PN 323110) and RNAscope^®^ wash buffer (PN 310091) (Advanced Cell Diagnostics, USA) was used to detect target genes according to manufacturer recommendations.

### 4.17 Immunofluorescence staining

Bake slides of patient tissues were washed with PBS, permeabilised with 0.3% Triton X‐100 in PBS, and then blocked in 5% BSA followed by overnight incubation with primary antibodies at 4°C. The primary antibodies used for immunostaining were human anti‐CD3 (ab699, Abcam, UK), human anti‐CD19 (ab134114, Abcam, UK), and purified anti‐human TCR Vα7.2 (351702, Biolegend, USA). After washing, sections were incubated with secondary antibodies at room temperature for 2 h. Images were taken by an Olympus fluorescence microscope (BX61, OLYMPUS, Japan) and a confocal microscope (Lecia TCS SP8).

### 4.18 Statistical analysis

All statistical analyses and graph generation were performed in R (version 3.6.3). A Student's *t*‐test was used to analyse data.

## CONFLICT OF INTEREST

The authors have declared that no conflict of interest exists.

## Supporting information



Supporting information
