# Supplementary material for: Revealing the transcriptional heterogeneity of organ‐specific metastasis in human gastric cancer using single‐cell RNA Sequencing
Source: Clin Transl Med. 2022 Feb 20;12(2):e730. doi: 10.1002/ctm2.730 (PMC8858624; doi:10.1002/ctm2.730)
Supplement: Supplementary file 1 — Supporting information [file CTM2-12-e730-s001.pdf]

Supplementary Materials

(Figure S1 – S18 and Table S1 – S8)

Supplementary Methods

1.1 | Generating monocyte-derived macrophages and stimulated NK cells

Human peripheral blood mononuclear cells were purchased from OriBiotech (Shanghai, China). Monocytes and NK cells were isolated using human pan monocyte isolation kit and human NK cell isolation kit, respectively (Miltenyi Biotec, Bergisch Gladbach, Germany), following manufacturer's protocol. Fresh isolated monocytes were seeded into 96-cell plates (1×10^5 cells/well) and differentiated for 7 days with human rM-CSF (100 ng/ml; Novoprotein, Shanghai, China). Then, the monocyte-derived macrophages were obtained.[1] Freshly isolated NK cells were cultured with IL-2 (100 ng/ml; Novoprotein, Shanghai, China) for 24 h.

1.2 | Assessing interferon (IFN)- γ production

Stimulated NK cells were then seeded into 96-well plates (1×10^5 cells/well) and co-cultured with monocyte-derived macrophages for 24 h following anti-NKG2A antibody (1 μg/ml; MAB1059, R & D Systems, Minneapolis, USA) or isotype-matched control antibody (1 μg/ml; MAB0031, R & D Systems, Minneapolis, USA) treatment. The IFN-γ levels in the culture supernatants were examined using enzyme-linked immunosorbent assay (ELISA) kits according to the manufacturers' instructions (KHC4021, Thermo Fisher Scientific, Massachusetts, USA).

Supplementary Figures

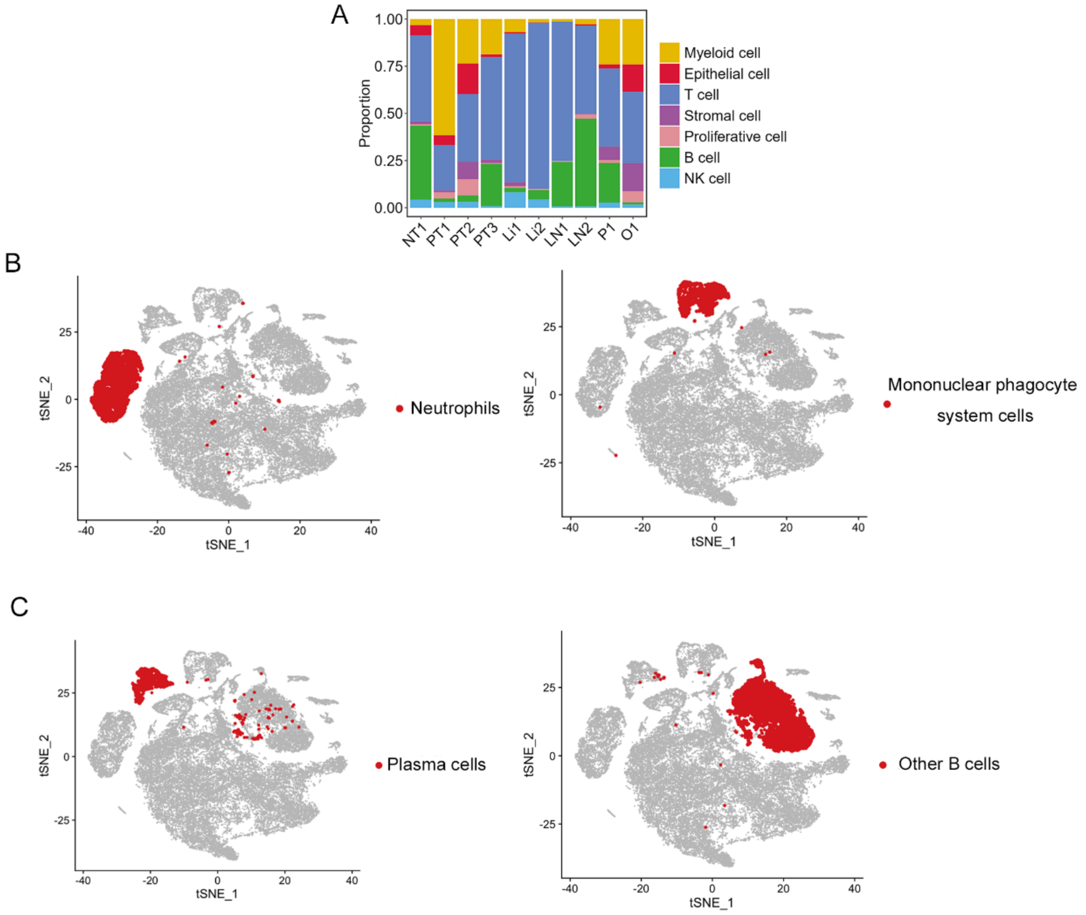

FIGURE S1 (A) Relative proportion of different cell clusters from the tissues of each origin. The relative proportion of cell subsets from tissues of each origin. NT1: the adjacent non-tumor sample; PT1, PT2, PT3: the primary tumor samples; Li1, Li2: the liver metastasis samples; LN1, LN2: the lymph node metastasis samples; P1: the peritoneum metastasis sample; O1: the ovary metastasis sample. (B) Neutrophils and mononuclear phagocyte system cells shown in tSEN of all cells. (C) Plasma cells and other B cells shown in tSEN of all cells.

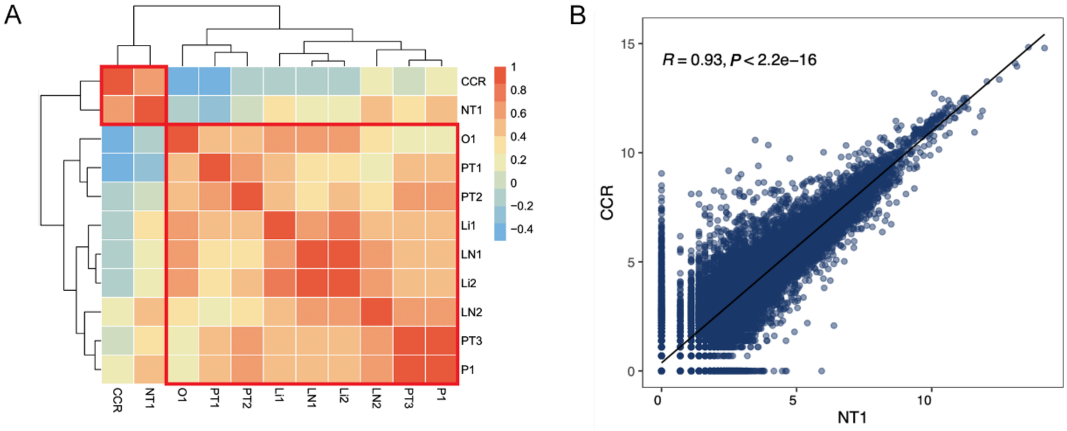

FIGURE S2 The correlation coefficient between the NT1 and the three GC normal tissues from a previously published scRNA-seq study.[2] (A) Heatmap showing the spearman correlation between all the samples in this study and the three GC normal tissues based on the top 20 DEGs between normal and tumor samples. CCR: three GC normal tissues from a previously published scRNA-seq study.[2] (B) Scatterplot showing the Pearson correlation of gene expression levels detected in NT1 and the three GC normal tissues.

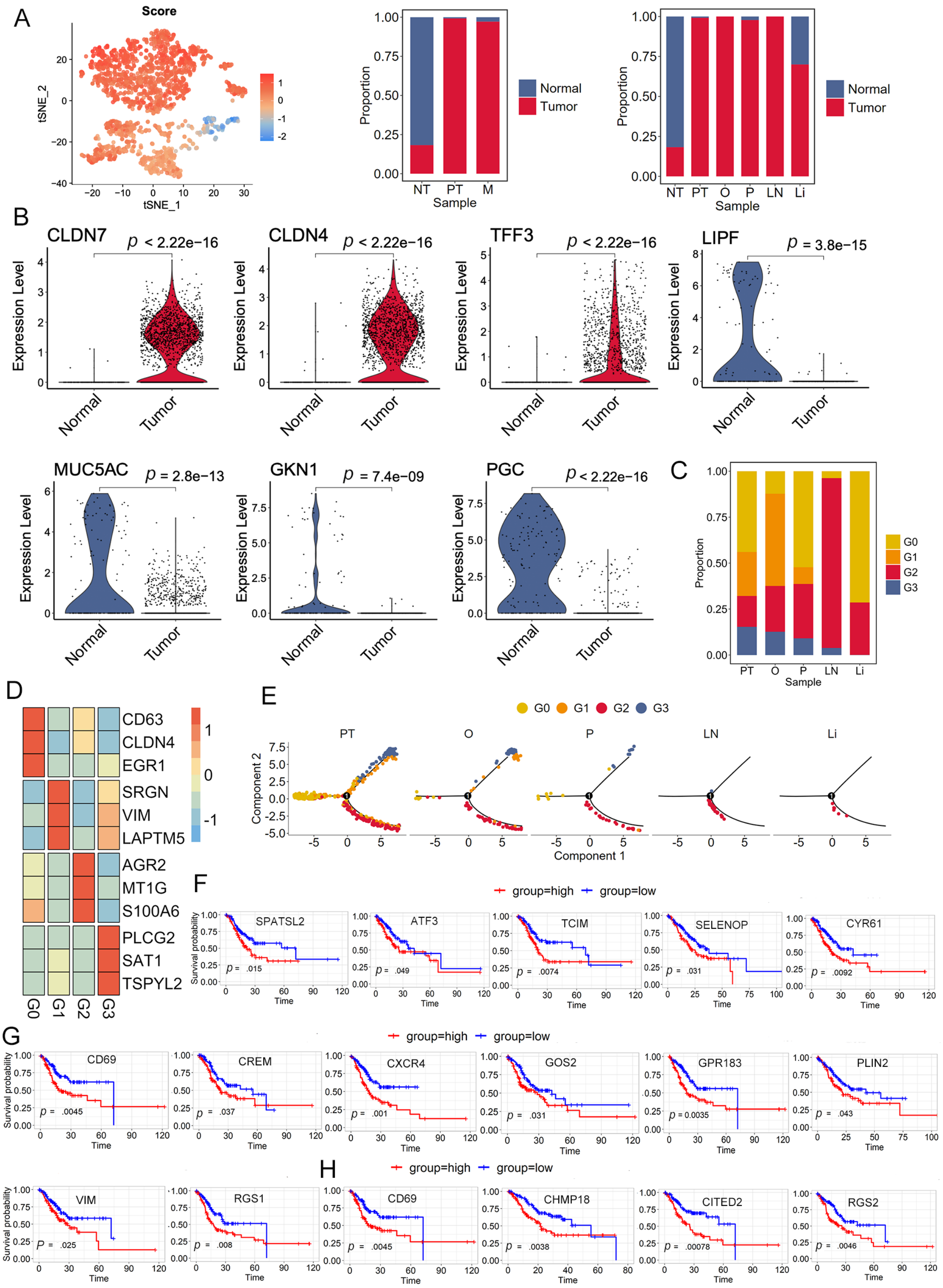

FIGURE S3 Analysis of normal and malignant epithelial cell subsets. (A) tSNE of 1,743 epithelial cells, colored according to malignant score minus non-malignant score. The relative proportion of cell subsets from tissues of each origin. NT: the adjacent non-tumor sample; PT: the primary tumor samples; M: all the metastasis samples; Li: the liver metastasis samples; LN: the lymph node metastasis samples; P: the peritoneum

metastasis sample; O: the ovary metastasis sample. (B) Violin plots showing the expression of seven representative genes within the differentially expressed genes (DEGs) between malignant and non-malignant cells. (C) The relative proportion of G0–G3 in NT, PT, O, P, LN, and Li samples. (D) Heatmap of marker genes in G0–G3 clusters. (E) Unsupervised transcriptional trajectory of G0–G3 in NT, PT, O, P, LN, and Li samples predicted by monocle 2. (F–G) High level of G0-associated genes (*TCIM*, *CYR61*, *SPATS2L*, *ATF3*, and *SELENOP*), G1-associated genes (*CD69*, *CREM*, *CXCR4*, *GOS2*, *GPR183*, *PLIN2*, *RGS1*, and *VIM*), and G3-associated genes (*CD69*, *CHMP1B*, *CITED2*, and *RGS2*) predicted poor prognosis in TCGA - STAD.htseq_counts.tsv dataset (*n* = 407 samples). Log-rank $p < .05$ was considered as statistically significant.

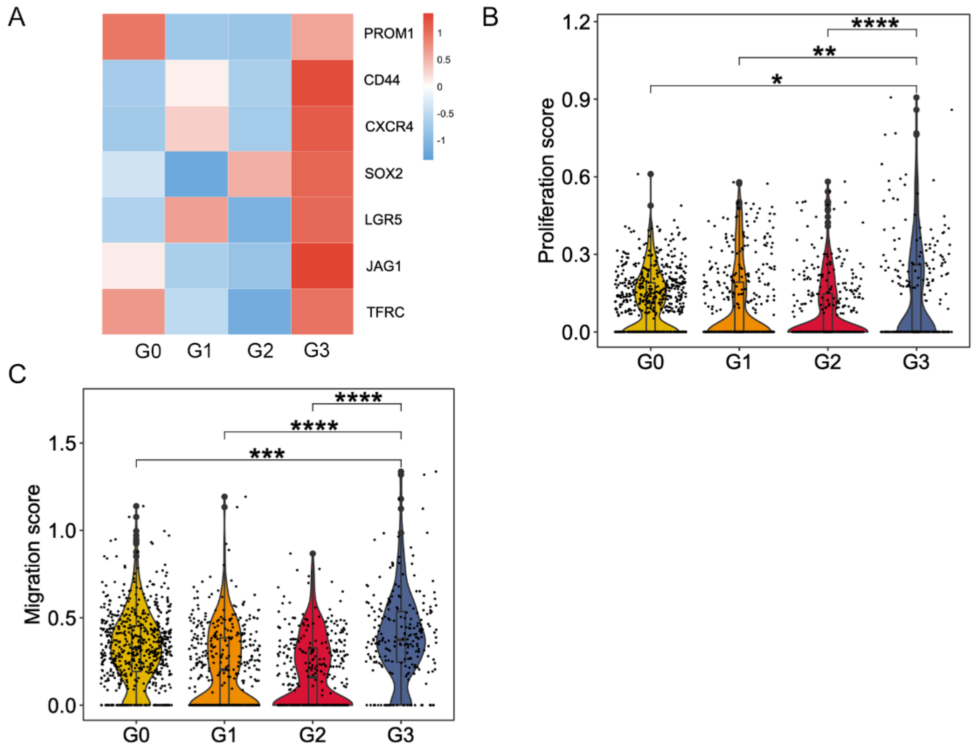

FIGURE S4 G3 cells might be EMT-induced CSCs and have higher proliferative and migrative capacity. (A) Heatmap showing the average expression of *CD44*, *PROM1*, *LGR5*, *SOX2*, *TFRC*, *CXCR4*, and *JAG1* in G0–G3 subclusters. (B–C) Violin plots showing the proliferation score (B) and migration score (C) of G0–G3 subcluster. $^{*}p < .05$, $^{**}p < .01$, $^{***}p < .001$, $^{****}p < .0001$.

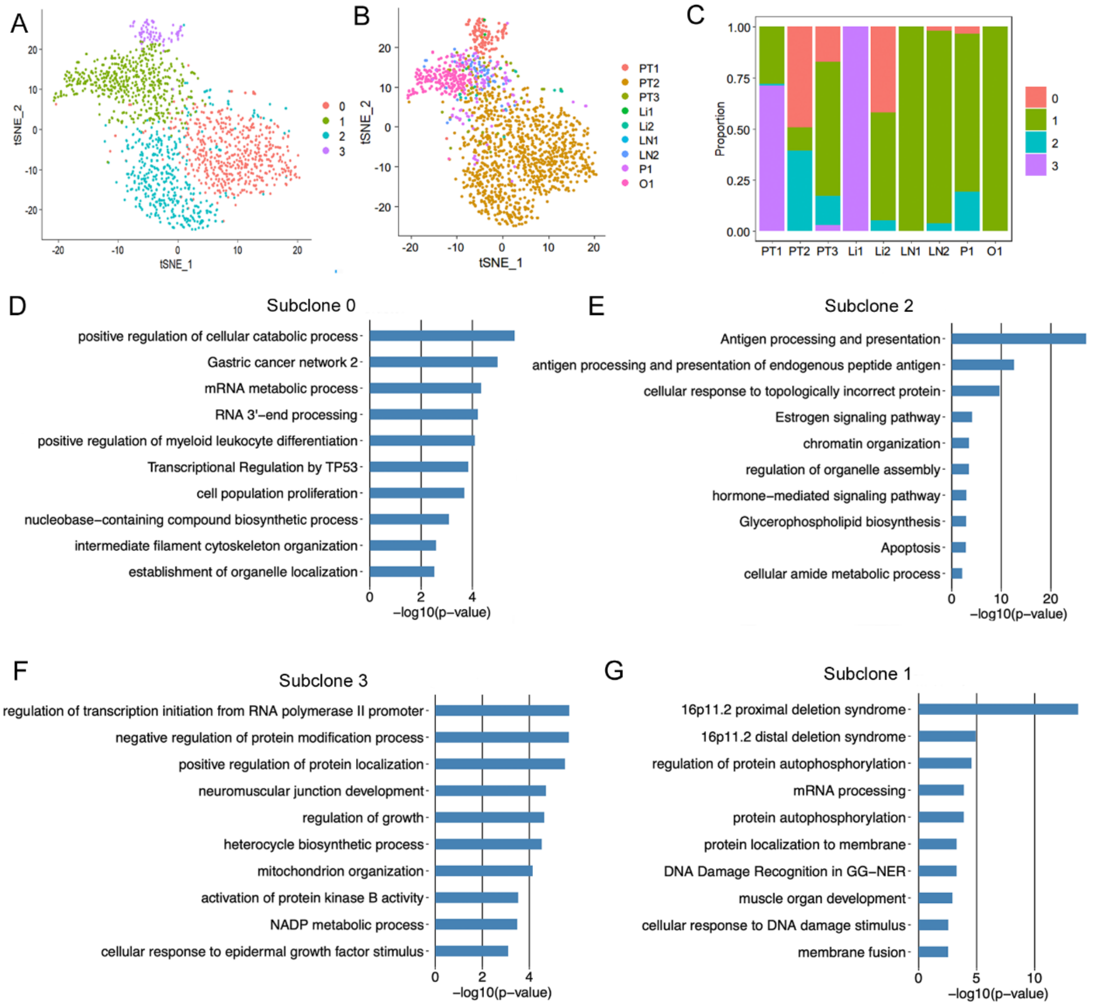

FIGURE S5 The CNVs in malignant epithelial cells showed inter- and intra-tumor heterogeneity between GC primary tumor and metastasis. (A) t-SEN of the four major subclones in malignant epithelial cells. (B) t-SEN of CNVs in PT (PT1, PT2, PT3), Li (Li1, Li2), LN (LN1, LN2), P (P1), and O (O1). (C) The relative proportion of four major subclones in PT (PT1, PT2, PT3), Li (Li1, Li2), LN (LN1, LN2), P (P1), and O (O1). (D) GO analysis of the top 10 pathways in subclone 0. (E) GO analysis of the top 10 pathways in subclone 2. (F) GO analysis of the top 10 pathways in subclone 3. (G) GO analysis of the top 10 pathways in subclone 1.

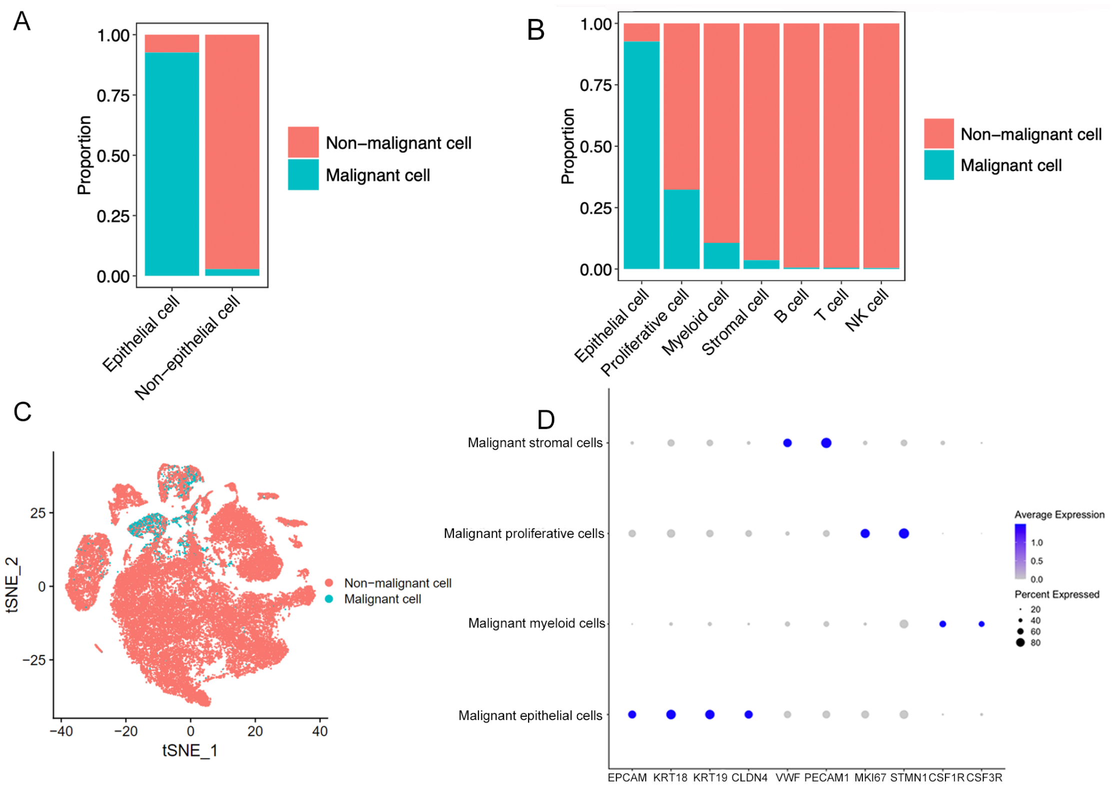

FIGURE S6 Analysis of malignant and non-malignant cells on non-epithelial cells. (A) The relative proportion of malignant and non-malignant cells on epithelial cells and non-epithelial cells. Since the cell type-specific marker gene interferes with the calculation of non-malignant score and malignant score during the iterative process, we only assigned cell identity (malignant cell or non-malignant cell) once on non-epithelial cells. (B) The relative proportion of malignant and non-malignant cells on different cell types. (C) tSNE of all cells, colored according to malignant and non-malignant cells. (D) Dot plot showing the canonical marker genes in stromal malignant cells, proliferative malignant cells, myeloid malignant cells, and epithelial malignant cells.

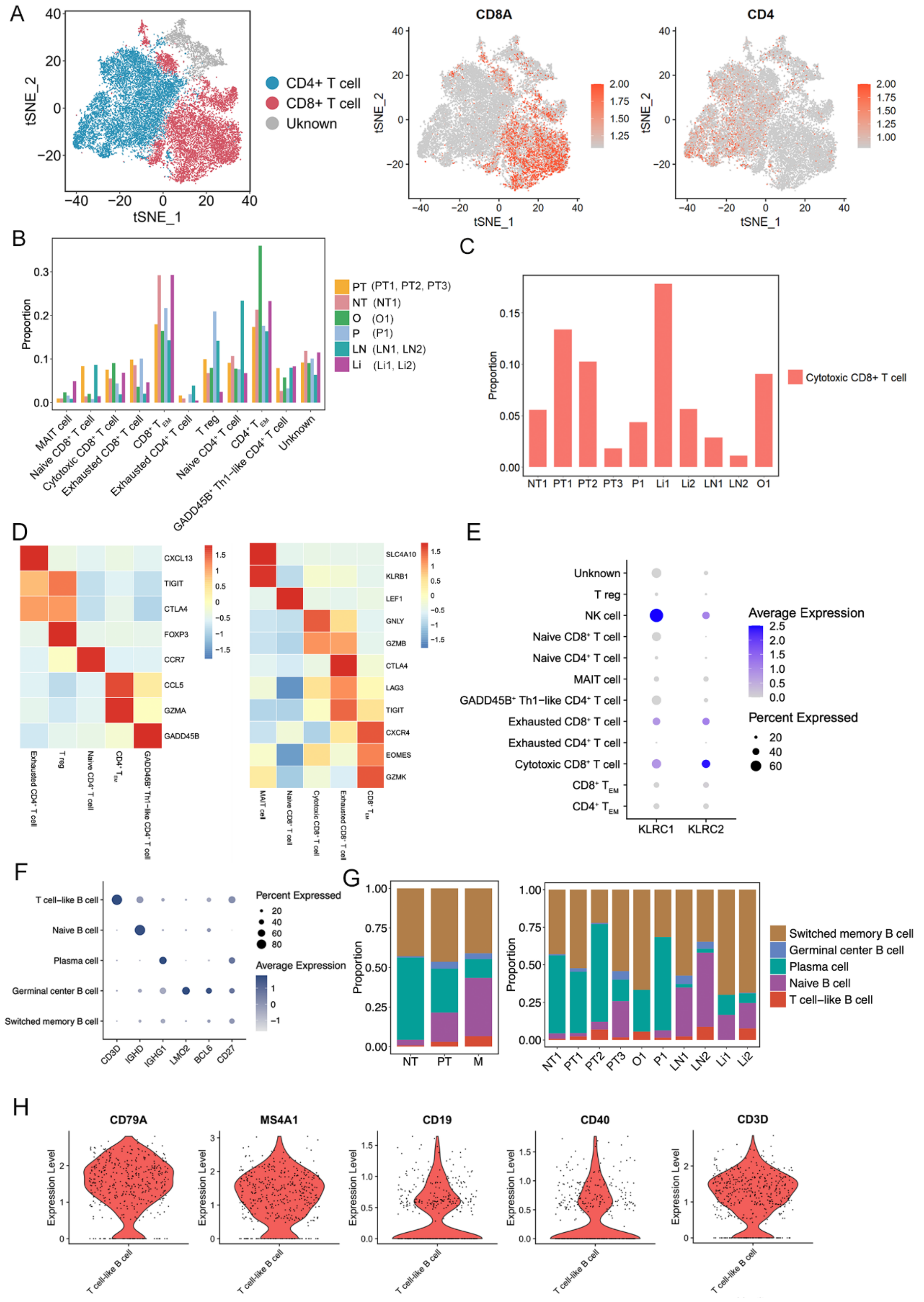

FIGURE S7 T cell and B cell subclusters. (A) tSNE of CD4$^+$ T cells and CD8$^+$ T cells, color-coded by marker genes as indicated. (B) The relative proportion of each T cell cluster in the NT (NT1), PT (PT1, PT2, PT3), Li (Li1, Li2), LN (LN1, LN2), P (P1),

and O (O1) samples. Tregs: regulatory T cells; CD4$^+$ T_{EM}: effector memory CD4$^+$ T cells; GADD45B$^+$ Th1-like CD4$^+$ T cells: GADD45B$^+$ T helper type 1-like CD4$^+$ T cells; CD8$^+$ T_{EM}: effector memory CD8$^+$ T cells; MAIT: mucosal-associated invariant T cells. (C) The relative proportion of cytotoxic CD8$^+$ T cells in NT1, PT1, PT2, PT3, Li1, Li2, LN1, LN2, P1, and O1 samples. (D) Heatmap of marker genes in each T cell cluster. (E) Dot plot showing the expression of *KLRC1* and *KLRC2* in T and NK cell clusters. (F) Dot plot showing the marker genes in each B cell cluster. (G) The relative proportion of each B cell cluster in NT (NT1), PT (PT1, PT2, PT3), M, Li (Li1, Li2), LN (LN1, LN2), P (P1), and O (O1). (H) Violin plot showing the expression of *CD79A*, *MS4A1*, *CD19*, *CD40*, and *CD3* on T cell-like B cells.

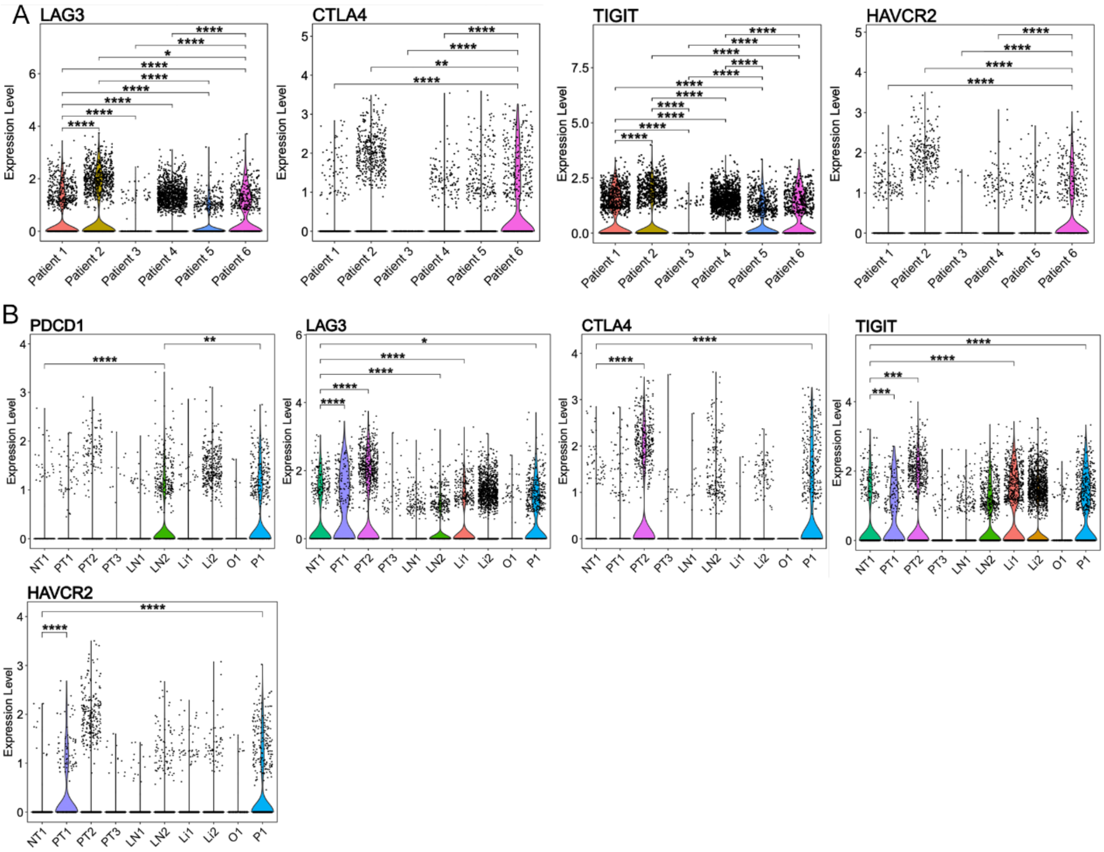

FIGURE S8 Immune-checkpoint molecules *PDCD1*, *LAG3*, *CTLA4*, *TIGIT*, and *HAVCR2* displayed in different patients and samples. (A) Violin plot showing the expression of *LAG3*, *CTLA4*, *TIGIT*, and *HAVCR2* on CD8$^+$ T cells across different patients. $^{*}p < .05$, $^{**}p < .01$, $^{****}p < .0001$. (B) Violin plot showing the expression of *PDCD1*, *LAG3*, *CTLA4*, *TIGIT*, and *HAVCR2* on CD8$^+$ T cells across different samples. $^{*}p < 0.05$, $^{***}p < .001$, $^{****}p < .0001$.

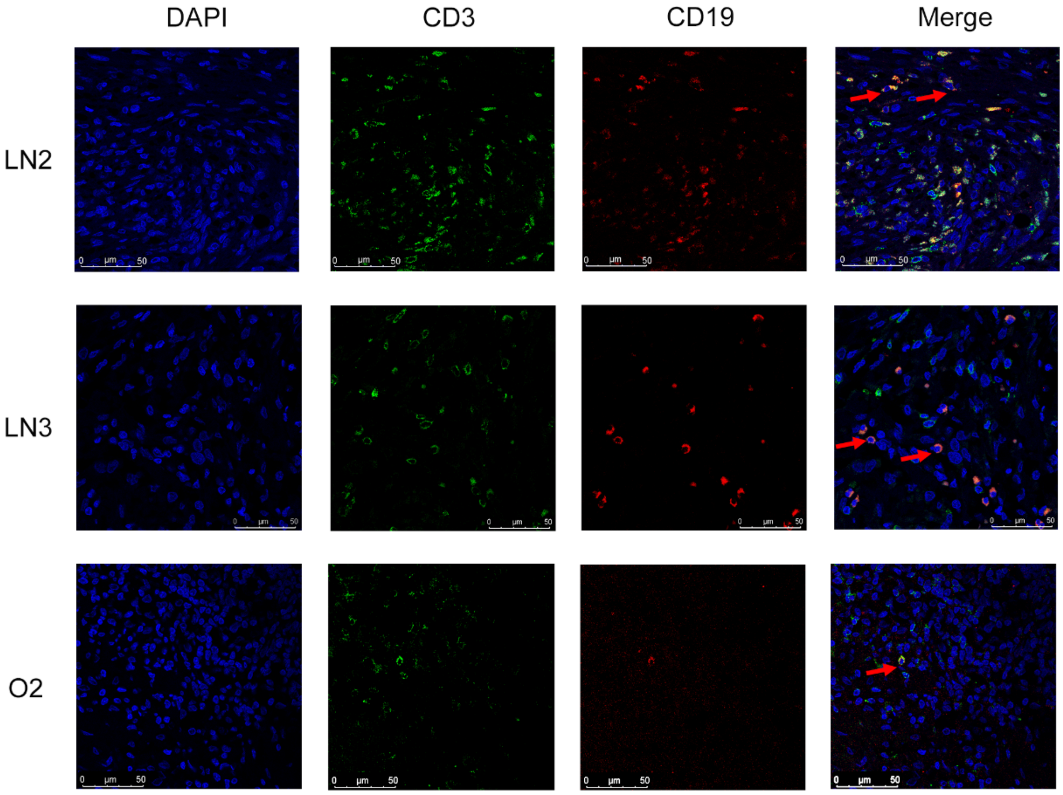

FIGURE S9 Immunofluorescence staining indicates the co-expression of CD19, CD3 and DAPI (nuclei) on T cell-like B cells in LN2, LN3, and O2. LN3 from the patient who had GC with lymph node metastasis; O2 from the patient who had GC with ovary metastasis.

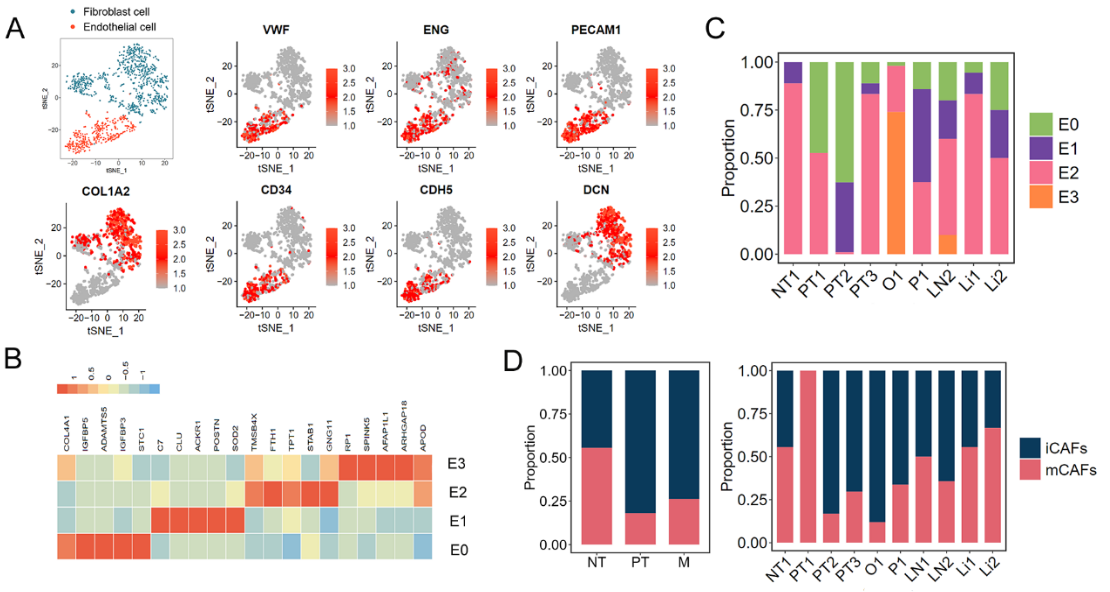

FIGURE S10 Endothelial cell and fibroblast subsets. (A) tSNE of endothelial cells and fibroblasts, color-coded by marker genes as indicated. (B) Heatmap of marker genes in E0–E3 cluster. (C) The relative proportion of each endothelial cell cluster (E0-E3) in NT(NT1), PT (PT1, PT2, PT3), M, Li (Li1, Li2), LN (LN1, LN2), P (P1), and O (O1). (D) The relative proportion of each fibroblast cell cluster (iCAFs and mCAFs) in NT(NT1), PT (PT1, PT2, PT3), M, Li (Li1, Li2), LN (LN1, LN2), P (P1), and O (O1).

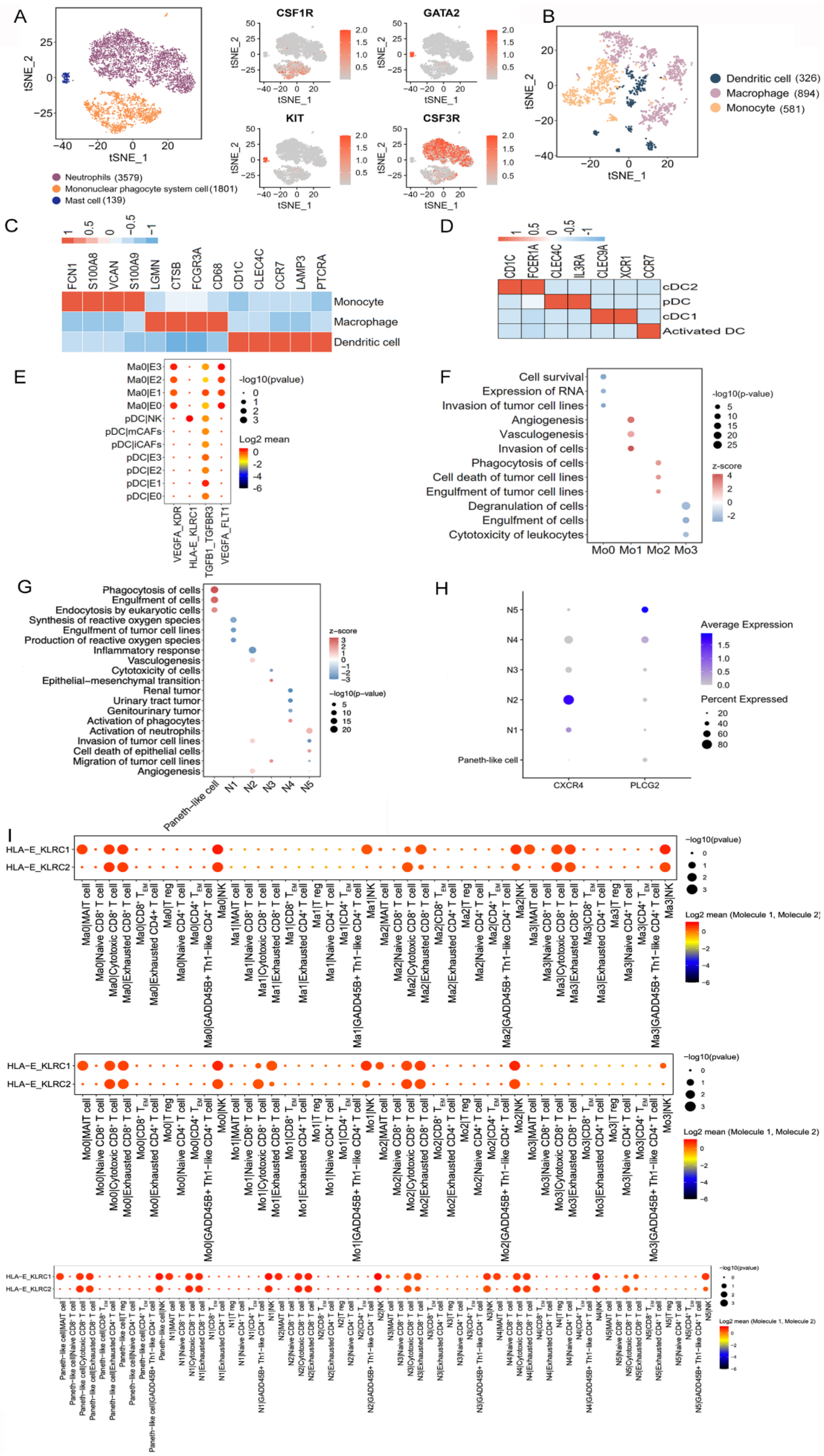

FIGURE S11 Myeloid cell subsets. (A) tSNE of mononuclear phagocyte system cells, mast cells, and neutrophils, color-coded by marker genes as indicated. (B) tSNE of DCs,

macrophages, and monocytes. (C) Heatmap of marker genes in DCs, macrophages, and monocytes. (D) Heatmap of marker genes in DCs clusters. (E) Bubble plots exhibiting significant interactions between macrophage cluster Ma0 and endothelial cells and between pDCs and other cell groups by the ligand-receptor pairs. (F) Dot plot showing the diseases and bio functions in monocytes (Mo0-Mo3) based on the DEGs by IPA. (G) Dot plot showing the diseases and bio functions in each neutrophil cluster based on the DEGs by IPA analysis. (H) Dot plot showing the expression of *CXCR4* and *PLCG2* in each neutrophil cluster. (I) Bubble plots exhibiting significant interactions between macrophages and T/NK cells, monocytes and T/NK cells, neutrophils and T/NK cells by the ligand-receptor pairs.

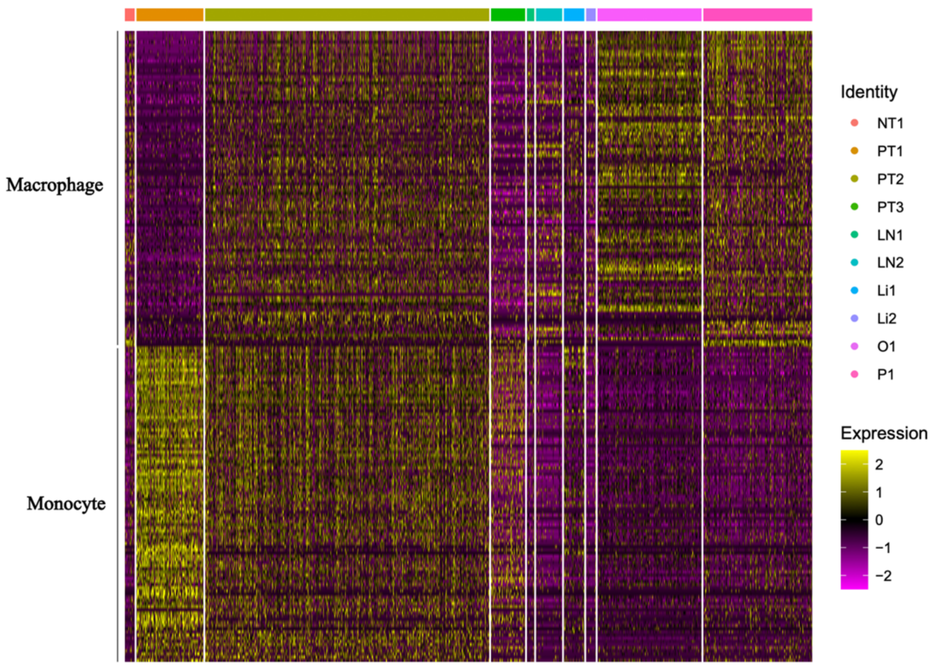

FIGURE S12 Heatmap showing transcriptional differences between macrophages and monocytes in the same sample (NT1, PT1, PT2, PT3, Li1, Li2, LN1, LN2, O1, and P1), which was based on the DEGs between all the macrophages and monocytes.

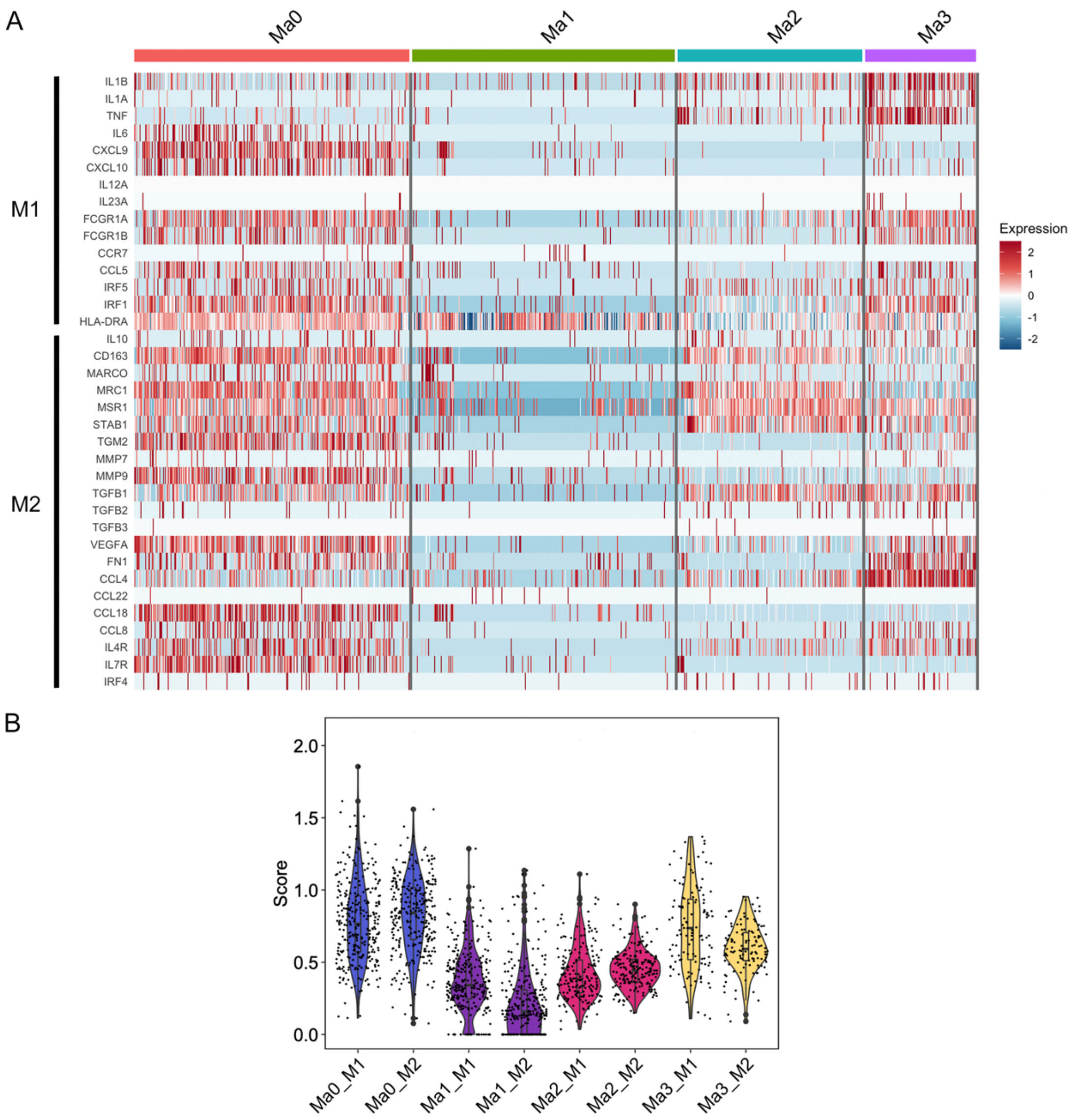

FIGURE S13 Ma1 and Ma3 cells are more inclined to have M1 signature. (A) Heatmap describing the expression of M1 and M2 feature genes in Ma0–Ma3. (B) Violin plot showing the mean score of the M1 or M2 signature across Ma0–Ma3.

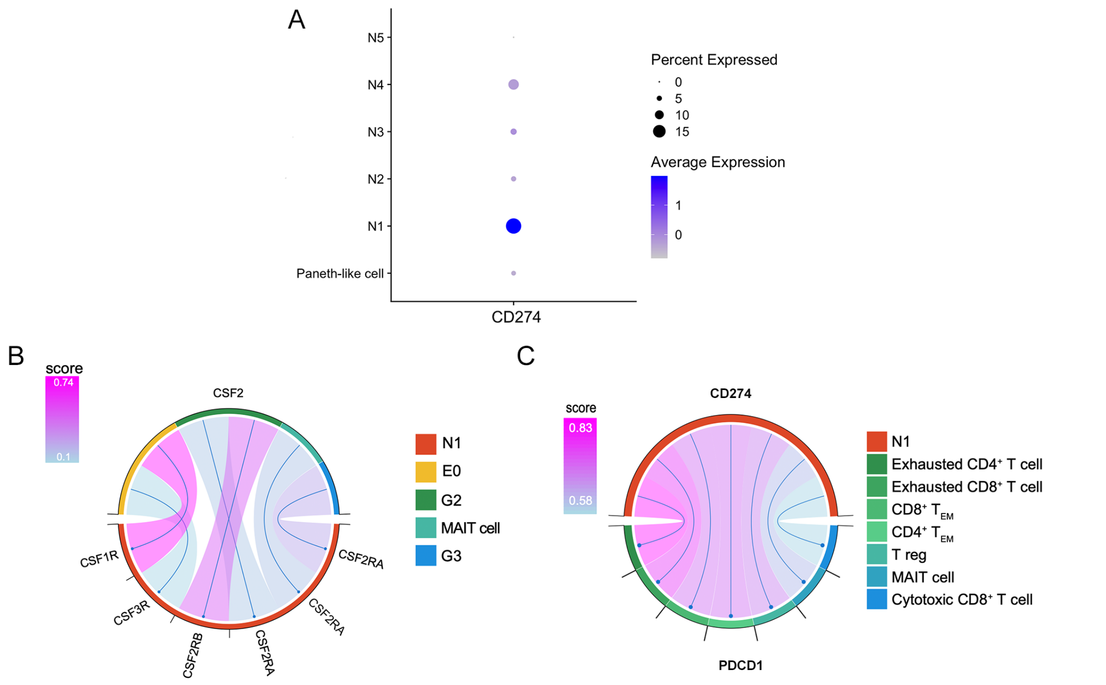

FIGURE S14 N1 cells express *PD-L1* (*CD274*) and suppress T cell immunity through PD-L1/PD-1 interaction. (A) Dot plot showing the average expression of *PD-L1* (*CD274*) in neutrophils subclusters. (B) Chord diagram showing N1 cells may receive GM-CSF from G2, G3, E0, and MAIT cells through GM-CSF (CSF2) and its receptor CSF2RA/CSF2RB/CSF1R/CSF3R. (C) Chord diagram showing the ligand-receptor pair PD-L1/PD-1 involved in interactions between N1 and T-cell subclusters.

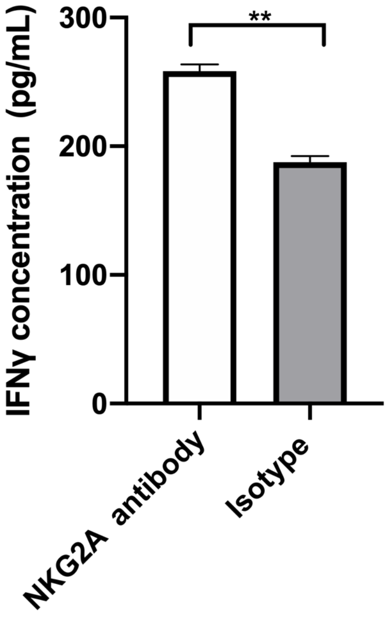

FIGURE S15 IFNγ levels in the co-culture of monocyte-derived macrophages and NK cells following either anti-NKG2A antibody blockade or isotype. $^{**}p < .01$, data represent with median ± SD, $n = 2$.

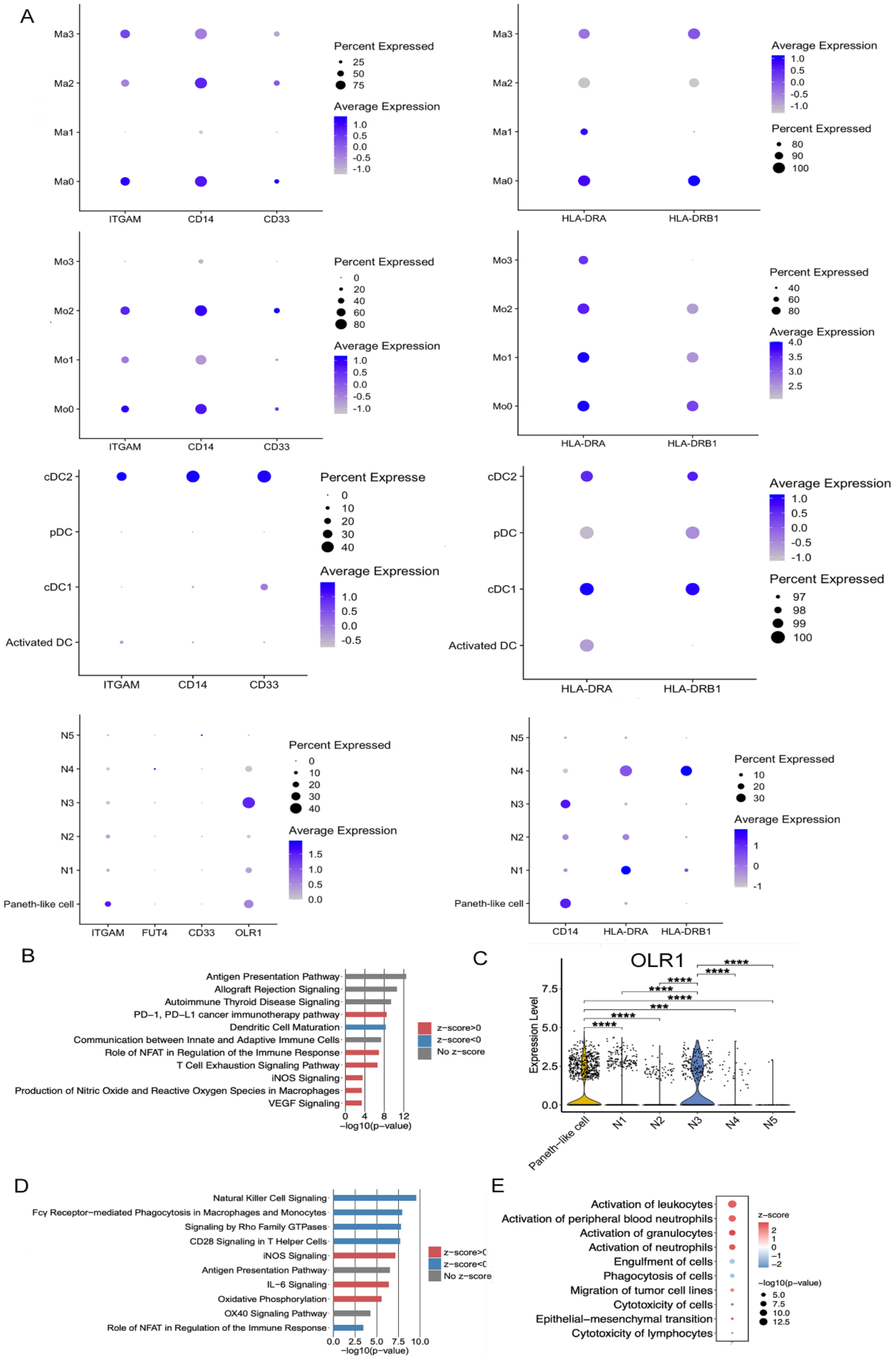

FIGURE S16 Ma2 cells were identified as M-MDSCs and N3 cells were identified as G-MDSCs. (A) Dot plot showing the average expression of the marker genes of

MDSCs in macrophage, monocyte, neutrophil, and DC clusters. (B) IPA results showing the top enriched canonical pathways in Ma2 cells based on the DEGs between Ma2 and the other macrophages. z-score > 0 represents the pathway is activated; z-score < 0 means the pathway is inhibited; no z-score indicates the pathway cannot be predicted whether it is activated or inhibited. (C) Violin plot showing the expression level of *OLR1* in neutrophil clusters. $^{**}p < .01$, $^{****}p < .0001$. (D) IPA results showing the top enriched canonical pathways in N3 cells based on the DEGs between N3 and the other neutrophils. z-score > 0 represents the pathway is activated; z-score < 0 means the pathway is inhibited; no z-score indicates the pathway cannot be predicted whether it is activated or inhibited. (E) Dot plot showing the diseases and bio functions in N3 cells based on the DEGs between N3 and the other neutrophils. z-score > 0 represents the disease and bio function is activated; z-score < 0 means the disease and bio function is inhibited.

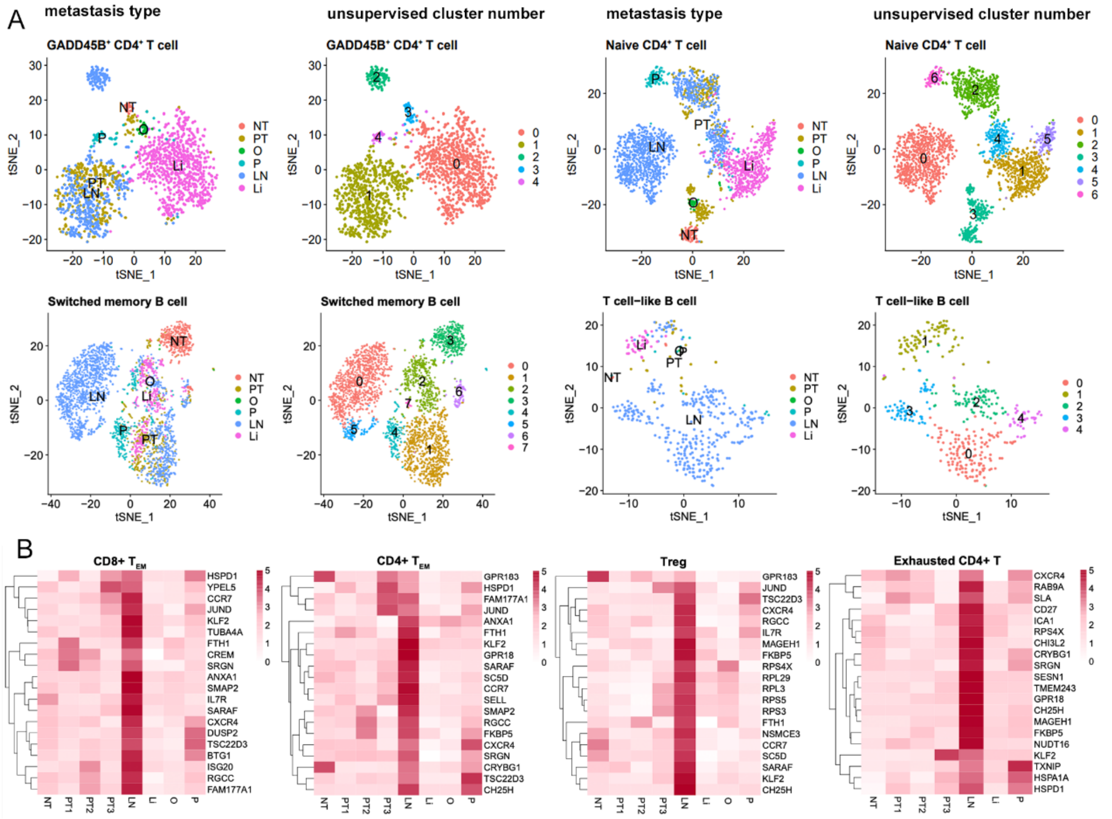

FIGURE S17 Different metastasis types populated different T and B lymphocyte subclusters. (A) tSNE of GADD45B Th1-like CD4$^+$ T cells, naive CD4$^+$ T cells, T-cell like B cells, and switch memory B cells colored with metastasis type and unsupervised cluster number. (B) Heatmap of top 20 DEGs in CD8$^+$ T_{EM}, CD4$^+$ T_{EM}, Tregs and exhausted CD4$^+$ T cells in the different samples.

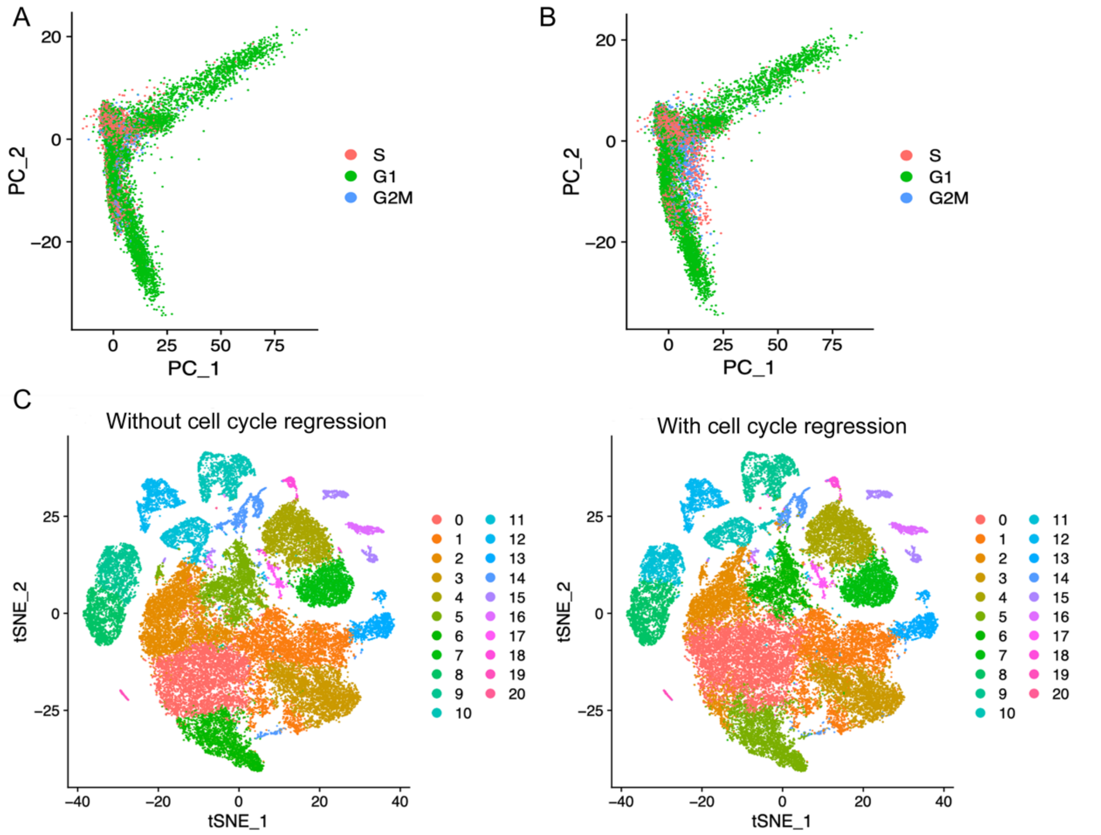

FIGURE S18 Cell cycle effects in the analysis. (A) Principal Component Analysis (PCA) showing the cell cycle scores of all the cells without cell cycle regression. (B) PCA showing the cell cycle scores of all the cells with cell cycle regression. (C) tSEN showing the results of cell clustering with or without cell cycle regression.

Supplementary Tables

Table S1 Clinical characteristics of samples used in scRNA-seq study.

Patient	Sample	Age	Sex	Blood type	Lauren's classification	Collect ways	Histopathological diagnosis	Number of cells
Patient 1	PT1	60s	Female	O	Intestinal	Gastroscope	Distal gastric adenocarcinoma with liver metastasis	2,866
	Li1					Liver biopsy		1,348
Patient 2	PT2	80s	Female	O	Intestinal	Operation	Moderately differentiated gastric adenocarcinoma, no metastasis	9,226
	NT1							2,777
Patient 3	O1	40s	Female	B	Mixed	Ovarian puncture	Poorly differentiated gastric adenocarcinoma with ovary metastasis	5,937
Patient 4	PT3	60s	Male	O	Intestinal	Operation	Moderately differentiated gastric adenocarcinoma with liver and lymph node metastases	3,925
	Li2							11,164
	LN1							4,227
Patient 5	LN2	80s	Female	B	Mixed	Operation	Moderately poorly differentiated gastric adenocarcinoma with lymph node metastasis	7,940
Patient 6	P1	70s	Male	A	Intestinal	Operation	Moderately poorly differentiated gastric adenocarcinoma with peritoneum metastasis	5,277

Table S2 Canonical pathway analysis using IPA.

Cluster	Pathways	-log(p-value)	zScore	Ratio	Molecules
**G0**	ILK Signaling	5.50	2.713	0.065	FOS, ITGB1, ITGB8, KRT18, MUC1, MYL9, PIK3R1, RHOB, RND3, TMSB10, TMSB4X, VEGFA, VIM
**G0**	IL-8 Signaling	3.80	2.333	0.051	CXCL1, FOS, ICAM1, ITGAV, MYL9, NCF1, PIK3R1, RHOB, RND3, VEGFA
**G2**	p53 Signaling	6.02	1.134	0.092	CDKN2A, GADD45A, GADD45B, JMY, JUN, MDM2, PIK3R1, PMAIP1, SERPINB5
**G3**	SPINK1 General Cancer Pathway	5.10	1.890	0.131	MT1F, MT1G, MT1H, MT2A, PIK3R1, PRSS3, RASD1, SPINK1
**G3**	HGF Signaling	4.78	1.414	0.091	CDKN2A, DOCK1, ELF3, FOS, ITGB1, JUN, PIK3R1, PLCG2, RAC1, RASD1
**G3**	Regulation of The Epithelial Mesenchymal	4.07	1.732	0.064	CLDN3, FOS, ID2, JUN, PIK3R1, RAC1, RASD1, TNF, TNFSF10, TNFSF9, VIM, ZEB2

	Transition by Growth Factors Pathway				
**E1**	T Cell Exhaustion Signaling Pathway	10.70	1.134	0.107	BCL6, FOS, FOXO1, FOXP1, HLA-DMA, HLA-DMB, HLA-DPA1, HLA-DPB1, HLA-DQA1, HLA-DQB1, HLA-DRA, HLA-DRB1, HLA-DRB5, HLA-E, JUN, PPP2R5A, TGFBR2, TGFBR3
**E3**	White Adipose Tissue Browning Pathway	2.75	2.449	0.047	ADCY1, CACNG8, FGFR1, LDHB, NPR1, PRKAR2B

Table S3 Diseases and bio functions analysis using IPA.

cluster	Functions	Diseases or Functions Annotation	p-Value	Predicted Activation State	Activation z-score	Bias-corrected z-score	Up-regulated molecules
**G1**	mammary tumor	Mammary tumor	3.42E-13	Increased	2.213	2.347	**AREG, ARID5B,** CCR7, CD44, CD69, CXCR4, DUSP2, G0S2, GPR183, HLA-DQA1, IL32, KNL1, LGALS1, NR4A2, PGAP1, PIK3R1, PLIN2, PTGER4, RGS1, S100A4, SELL, TNFRSF1B
**G1**	genitourinary tumor	Genitourinary tumor	2.03E-09	Increased	2.213	2.263	**AREG, ARID5B, ARL4C,** BCAS2, CA2, LAPTM5, CD2, CCR7, CD44, CD69, CD79A, CKAP2, CREM, CXCR4, DUSP2, EIF4A3, G0S2, GCNT1,

							GIMAP7, GPR183, HLA-DQA1, HLA-DQB1, HMGB2, HSPH1, IGHM, IL32, ITM2A, KLF2, IL7R, KNL1, LTB, LGALST, MAL, MCM5, NFKBIA, NR4A2, PGAP1, PIK3R1, PTGER4, PLIN2, RGCC, RGS1, S100A4, SAMSN1, SDCBP, SELL, SESN3, SLA, SLC12A2, TNFRSF1B, VIM
**G1**	extracranial solid tumor	Extracranial solid tumor	2.11E-07	Increased	2.577	2.762	**AREG, ARID5B, ARL4C,** ATP1B3, BASP1, BATF, BCAS2, CA2, CCR7, CD2, CD27, CD44, CD7, CD69, CD79A, CKAP2, CLEC2B, COTL1, CREM, CXCR4, DUSP2, EIF4A3, G0S2, GCNT1, GIMAP7, GPR18,

	GPR183, HLA-DQA1, HLA-DQB1, HMGB2, HSPH1, IGHM, IL32, IL7R, ITM2A, KLF2, KNL1, LAPTM5, LGALS1, LIMS1, LTB, MAL, MANF, MCM5, NCF1, NFKBIA, NR4A2, NUCB2, PGAP1, PIK3R1, PLIN2, PTGER4, RGCC, RGS1, RILPL2, S100A4, SAMHD1, SAMSN1, SDCBP, SELL, SESN3, SLA, SLC12A2, SPCS1, SRGN, TNFAIP3, TNFRSF1B, VIM, ZNF331

Table S4 Patient medication information.

Patient	Running medications	Prognosis of patients
Patient 1	cTxNxM1, Phase IV<br>2019-11-12 to 2020-2-21: day 1 and day 8 Albumin-bound paclitaxel 125 mg/m^2, + day 1 Trastuzumab 8 mg/m^2+ day 1–day 14 S-1 40 mg/bid, q3w.<br>2020-3-13 to date: day 1–day 14 S-1 40mg/bid, q3w.	Stable disease
Patient 2	2019-11-5: Radical gastrectomy, pT3N0M0, Phase IIA.<br>2019-12-1 to 2020-2-1: day 1–day 14 S-1 40 mg/bid, q3w.	Disease free status
Patient 3	cTxNxM1, Phase IV<br>2019-12-6 to 2020-3-19: day 1 Oxaliplatin 130 mg/m^2 + day 1–day 14 Capecitabine 1500 mg/bid + day 1 Sintilimab/placebo 3 mg/kg, q3w.<br>2020-4-9 to 2020-5-20: day 1 Albumin-bound paclitaxel 125 mg/m^2, + day 1–day 7 S-1 60 mg/bid + day 1 Camrelizumab 200 mg, q2w.<br>2020-6-4 to 2020-7-14: day 1 Irinotecan 180 mg/m^2 + day 1 Camrelizumab 200 mg + Apatinib 250 mg qd, q2w.	Disease progression

	2020-8-6: day 1 docetaxel 50 mg + day 1 Camrelizumab 200 mg + Apatinib 250 mg qd, q3w.<br>2020-8-27 to date: Apatinib 250 mg qd + traditional Chinese medicine.	
Patient 4	2019-12-3: Radical gastrectomy, pT4aN3aM1, Phase IV.<br>No medical treatment.	Disease free status
Patient 5	2019-12-19: Radical gastrectomy, pT3N3aM0, Phase IIIB.<br>No medical treatment.	Disease free status
Patient 6	cT4aNxM1, Phase IV<br>2020-1-6 to 2020-2-24: day 1–day 14 S-1 40 mg/bid + day 1 Camrelizumab 200 mg, q3w.	Stable disease

Table S5 Top 50 highly expressed genes in tumor tissues and top 50 highly expressed genes in normal tissues from TCGA-STAD.htseq_counts.tsv.

Number	**Gene**	**Group**	**logFC**	**adj.P.Val**
1	IBSP	tumor	2.47	6.02E-10
2	CST4	tumor	2.39	8.26E-14
3	HOXC9	tumor	2.27	1.15E-10
4	FEZF1	tumor	2.27	3.89E-07
5	HOXA11	tumor	2.00	5.40E-05
6	RDH8	tumor	1.96	1.06E-09
7	ALPP	tumor	1.95	1.82E-07
8	HOXC10	tumor	1.86	9.12E-04
9	MAGEA12	tumor	1.82	3.20E-05
10	TAS2R38	tumor	1.81	6.53E-09
11	EVX1	tumor	1.81	1.63E-07
12	CCL7	tumor	1.80	2.40E-10
13	C5orf46	tumor	1.80	3.17E-08
14	DMBX1	tumor	1.79	3.45E-08
15	HOXC13	tumor	1.77	1.36E-05
16	FGF19	tumor	1.75	1.36E-07

17	MAGEA6	tumor	1.74	2.66E-04
18	HOXC12	tumor	1.73	2.56E-05
19	R3HDML	tumor	1.72	3.50E-10
20	ALPG	tumor	1.72	4.24E-05
21	MMP8	tumor	1.71	5.08E-09
22	MAGEA3	tumor	1.69	5.18E-04
23	HOXC11	tumor	1.67	3.04E-04
24	PRAC2	tumor	1.66	8.48E-07
25	SP8	tumor	1.64	1.69E-06
26	DCSTAMP	tumor	1.62	1.70E-12
27	CST1	tumor	1.62	4.86E-03
28	PIWIL1	tumor	1.61	1.09E-05
29	KRTAP4-1	tumor	1.61	3.24E-05
30	CSF2	tumor	1.59	2.56E-09
31	CSAG3	tumor	1.59	6.80E-05
32	ACTL8	tumor	1.59	5.59E-06
33	HOXC8	tumor	1.57	3.61E-07
34	BAAT	tumor	1.56	9.54E-07
35	KLK8	tumor	1.56	4.90E-05

36	SPDYC	tumor	1.55	7.18E-08
37	CFAP47	tumor	1.53	3.16E-07
38	MC2R	tumor	1.51	2.32E-07
39	NT5DC4	tumor	1.50	7.41E-18
40	PKD2L1	tumor	1.50	1.17E-18
41	MSLNL	tumor	1.47	1.33E-06
42	KNG1	tumor	1.45	1.04E-07
43	HTRA4	tumor	1.45	3.19E-18
44	IL37	tumor	1.44	1.30E-09
45	ZNF280A	tumor	1.43	3.05E-05
46	UCN2	tumor	1.43	1.94E-08
47	ACTBL2	tumor	1.42	3.58E-08
48	PAEP	tumor	1.42	1.35E-06
49	BIRC7	tumor	1.42	5.56E-10
50	CT83	tumor	1.41	1.83E-03
51	SERTM1	normal	2.20	2.53E-52
52	ADH7	normal	2.00	6.47E-36
53	CWH43	normal	1.84	8.00E-26
54	AQP4	normal	1.79	1.73E-44

55	ADIPOQ	normal	1.72	2.25E-25
56	CIDEA	normal	1.66	7.46E-26
57	HTR1E	normal	1.60	2.20E-15
58	PGA4	normal	1.48	3.04E-10
59	CMTM5	normal	1.46	1.13E-32
60	WIF1	normal	1.46	9.45E-23
61	PGA3	normal	1.45	1.87E-17
62	PGA5	normal	1.43	1.94E-16
63	NKX6-2	normal	1.42	2.04E-31
64	FAM180B	normal	1.40	2.32E-22
65	LMX1A	normal	1.39	1.61E-11
66	KCTD8	normal	1.39	8.46E-18
67	VSTM2A	normal	1.34	5.49E-21
68	PMP2	normal	1.31	2.80E-23
69	SH3GL2	normal	1.30	8.95E-34
70	CMA1	normal	1.30	3.02E-28
71	SYT10	normal	1.30	7.51E-13
72	TRARG1	normal	1.28	1.42E-17
73	LGI1	normal	1.27	3.45E-30

74	SLC5A7	normal	1.26	1.19E-18
75	PCSK2	normal	1.24	1.16E-38
76	DCAF12L1	normal	1.24	7.57E-09
77	SLC7A3	normal	1.17	5.60E-16
78	GCG	normal	1.17	5.05E-06
79	CHIA	normal	1.15	2.01E-06
80	RPRM	normal	1.14	6.62E-27
81	PLP1	normal	1.13	1.44E-52
82	PTF1A	normal	1.13	3.32E-07
83	KIAA0408	normal	1.13	8.89E-11
84	NR0B1	normal	1.13	1.88E-09
85	PEBP4	normal	1.13	1.86E-28
86	CCKBR	normal	1.12	3.24E-21
87	LGI3	normal	1.12	5.48E-21
88	HSPB3	normal	1.12	2.23E-06
89	PLD5	normal	1.11	4.27E-16
90	CCKAR	normal	1.11	1.80E-08
91	SYT4	normal	1.10	9.55E-10
92	NUPR2	normal	1.10	4.17E-08

93	GKN1	normal	1.10	5.47E-14
94	ATP4B	normal	1.08	1.49E-12
95	NPPC	normal	1.08	7.71E-20
96	RXRG	normal	1.08	6.74E-34
97	GPR12	normal	1.08	1.68E-07
98	TMEM132C	normal	1.07	2.38E-31
99	FLG2	normal	1.07	8.35E-08
100	HS3ST6	normal	1.07	2.62E-10

Table S6 Overview of T cell cluster lineages and associated functions.

T cell cluster names	Representative genes	Functional properties
CCR7$^+$ CD4	*CCR7*, *SELL*, *CD55*, *GPR18*, *KLF2*, *PLAC8*	naïve CD4$^+$ T cell
FOXP3$^+$ CD4	*FOXP3, IL2RA, TNFRSF4*, *TNFRSF9, TNFRSF18*	regulatory T cell (Tregs)
TIGIT$^+$ CD4	*CTLA4*, *PDCD1*, *TIGIT*, *TNFRSF4*, *TOX2*, *ICA1*	exhausted CD4 T cells
GZMA$^+$ CD4	*KLRB1*, *CCL5*, *ANXA1*, *GZMK*, *GZMA*, *CCR6*, *IL7R*	effector memory CD4$^+$ T cells (CD4$^+$ T_{EM})
GADD45B$^+$ CD4	*FOS*, *GADD45B*, *EGR1*, *TNF*, *AREG*, *NR4A1*	GADD45B$^+$ T helper type 1 (Th1)-like CD4$^+$ T cells
LEF1$^+$ CD8	*CCR7*, *SELL*, *CD55*, *LEF1*, *TCF7*, *ZBTB10*	naïve CD8$^+$ T cells
GZMB$^+$ CD8	*GZMB*, *GNLY*, *KLRD1*, *NKG7*, *KLRF1*, *TYROBP*	effector memory CD8$^+$ T cells (CD8$^+$ T_{EM})
CTLA4$^+$ CD8	*CXCL13*, *CTLA4*, *ENTPD1*, *HAVCR2*, *TIGIT*, *PDCD1*	exhausted CD8$^+$ T cells
GZMK$^+$ CD8	*GZMK*, *CXCR4*, *ITM2C*, *EOMES*, *SH2D1A, CMC1*	cytotoxic CD8$^+$ T cells
SLC4A10$^+$ CD8	*KLRB1*, *IL7R*, *SLC4A10*, *HSPA6*, *KLRG1*, *CEBPD*	mucosal-associated invariant T (MAIT) cells

Table S7 Immune-checkpoint molecules displayed in different patients and samples.

Patient	Immune-checkpoint molecules	Sample	Immune-checkpoint molecules
Patient 1	*LAG3*, *TIGIT*	PT1	*LAG3*, *TIGIT*, *HAVCR2*
		Li1	*LAG3*, *TIGIT*
Patient 2	*LAG3*, *TIGIT*	PT2	*LAG3*, *TIGIT*, *CTLA4*
		NT1	*LAG3*, *TIGIT*
Patient 3	None	O1	None
Patient 4	None	PT3	None
		Li2	*TIGIT*
		LN1	None
Patient 5	*PDCD1*, *LAG3*, *TIGIT*	LN2	*PDCD1, LAG3*, *TIGIT*
Patient 6	*PDCD1*, *LAG3*, *TIGIT*, *CTLA4*, *HAVCR2*	P1	*PDCD1*, *LAG3*, *TIGIT*, *CTLA4*, *HAVCR2*

Table S8 GO enrichment analysis.

Cluster	Biological process	GeneRatio	BgRatio	pvalue	p.adjust	qvalue	geneID
**iCAFs**	extracellular matrix organization	41/97	368/18670	1.92E-44	4.58E-41	3.22E-41	DCN, MMP2, PDGFRA, LUM, MFAP2, SFRP2, COL11A1, PDPN, GREM1, COL10A1, COL8A1, CTSK, FBLN1, LRP1, COL6A3, SULF2, COL1A2, TIMP2, FAP, FBLN5, COL1A1, COL3A1, VCAN, LOX, COL5A1, MFAP5, EMILIN1, CCDC80, HTRA1, FBN1, COL7A1, POSTN, PLOD2, CYP1B1, SULF1, MMP14, ADAMTS2, SERPINE1, COL6A1, COL12A1, ANTXR1
**iCAFs**	extracellular matrix disassembly	8/97	80/18670	8.44E-09	5.61E-07	3.94E-07	MMP2, PDPN, CTSK, LRP1, TIMP2, FAP, HTRA1, MMP14

Reference

1. Camilli G, Cassotta A, Battella S, et al. Regulation and trafficking of the HLA-E molecules during monocyte-macrophage differentiation. *J Leukoc Biol.* 2016;99:121-130.
2. Sathe A, Grimes SM, Lau BT, et al. Single-Cell Genomic Characterization Reveals the Cellular Reprogramming of the Gastric Tumor Microenvironment. *Clin Cancer Res*. 2020;26:2640-2653.
